# Scandinavian guidelines for initial management of minimal, mild and moderate head injuries in adults: an evidence and consensus-based update

**DOI:** 10.1186/1741-7015-11-50

**Published:** 2013-02-25

**Authors:** Johan Undén, Tor Ingebrigtsen, Bertil Romner

**Affiliations:** 1 Department of Intensive Care and Perioperative Medicine, Institute for Clinical Sciences, Södra Förstadsgatan 101, 20502 Malmö, Sweden; 2 Department of Neurosurgery, Institute for Clinical Medicine, Sykehusveien 38, 9038 Tromsö, Norway; 3 Department of Neurosurgery, Institute for Clinical Medicine, Blegdamsvej 9, 2100 Copenhagen, Denmark

**Keywords:** computed tomography, GRADE, guidelines, head/brain injury/trauma, management, prediction rule, routines, S100/S100B/S100BB

## Abstract

**Background:**

The management of minimal, mild and moderate head injuries is still controversial. In 2000, the Scandinavian Neurotrauma Committee (SNC) presented evidence-based guidelines for initial management of these injuries. Since then, considerable new evidence has emerged.

**Methods:**

General methodology according to the Appraisal of Guidelines for Research and Evaluation (AGREE) II framework and the Grading of Recommendations Assessment, Development and Evaluation (GRADE) system. Systematic evidence-based review according to Preferred Reporting Items for Systematic Reviews and Meta-Analyses (PRISMA) methodology, based upon relevant clinical questions with respect to patient-important outcomes, including Quality Assessment of Diagnostic Accuracy Studies (QUADAS) and Centre of Evidence Based Medicine (CEBM) quality ratings. Based upon the results, GRADE recommendations, guidelines and discharge instructions were drafted. A modified Delphi approach was used for consensus and relevant clinical stakeholders were consulted.

**Conclusions:**

We present the updated SNC guidelines for initial management of minimal, mild and moderate head injury in adults including criteria for computed tomography (CT) scan selection, admission and discharge with suggestions for monitoring routines and discharge advice for patients. The guidelines are designed to primarily detect neurosurgical intervention with traumatic CT findings as a secondary goal. For elements lacking good evidence, such as in-hospital monitoring, routines were largely based on consensus. We suggest external validation of the guidelines before widespread clinical use is recommended.

## Background

Traumatic brain injury (TBI) is one of the most common reasons for emergency department (ED) care [1]. Cases of TBI account for over 1 million visits per year in both the USA and the UK [2,3] and are responsible for two-thirds of all trauma deaths [4]. Only a small proportion of these are classed as severe head injury [1], with a Glasgow Coma Scale (GCS) score of 3 to 8. The majority of patients are instead classed as minimal, mild and moderate head injuries [5] and are generally conscious in the ED with varying degrees of neurological symptoms. A minority of these patients will have intracranial pathology on computed tomography (CT) scanning and an even smaller proportion will need neurosurgical intervention [6,7]. In particular, the intermediate risk group of mild head injury (MHI) has been notoriously difficult to manage as these patients have a very low, but not negligible, risk of needing neurosurgical intervention [7,8].

Over the past decade, initial management strategies have become focused on selective CT use based upon presence or absence of specific aspects of patient history and/or clinical examination [6,9-11], in order to effectively use health care resources. This selective management has received more attention following reports of increased cancer risks from CT scans, estimated at 1 in 5,000 to 10,000 for a single head CT scan in young adults [12].

Following a normal CT scan after mild head injury, consensus is generally to discharge patients from the hospital [13,14], although subgroups of patients may still be at risk of developing delayed intracranial complications of varying significance [15,16].

In 2000, the Scandinavian Neurotrauma Committee (SNC) presented evidence-based guidelines for the initial management of minimal, mild and moderate head injuries [5]. Although external and independent validation has shown the guidelines to function favorably [17,18], it is likely that new evidence exists which needs to be considered. The SNC has therefore mandated an update of the guidelines. The aim of the present report is to present these updated guidelines for adults, including the methodology and considerations behind the workflow.

## Methods

**Figure 1:**
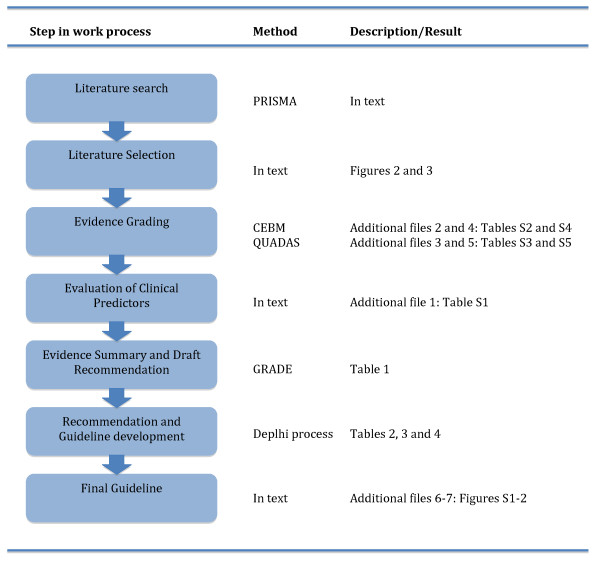
**Flow diagram showing the overall work process**.

The overall policy was to follow the Appraisal of Guidelines for Research and Evaluation (AGREE) II guideline development framework [19], complemented by the Grading of Recommendations Assessment, Development and Evaluation (GRADE) system [20]. Consensus was that these two aids would result in a transparent and systematic methodology and the best possible workflow from available evidence to guideline construction and implementation. The overall workflow process is shown in Figure 1.

### Task force, working group and stakeholders

The SNC consists of neurosurgeons and anesthesiologists from Scandinavia with expertise in neurotrauma. A task force was initiated within the SNC, consisting of three authors with experience within the field (JU, TI, BR), to propose evidence-based recommendations and a draft for the updated guidelines. For the consensus stage of development, a working group was formed consisting of SNC members. Important stakeholders from general surgery, emergency medicine and orthopedics were also involved in this process. These specialties initially manage the vast majority of head injury patients in Scandinavia. We also considered including members of the public in the process but unanimously decided against this as we did not believe it would facilitate optimal guideline development in the present scenario.

### Scope, purpose and target population

The objective of the guidelines created in the present work would be to assist ED physicians with initial (the first 24 h) management of all adult patients with minimal, mild and moderate head injury, specifically to decide which patients are to receive CT scanning, admission or discharge (or combinations of these) from the ED. Head injury severity was predefined according to the Head Injury Severity Score (HISS [21]) where minimal represents patients with a GCS score of 15 and no risk factors, mild is a GCS score of 14 or 15 with risk factors (such as amnesia or loss of consciousness (LOC)) and moderate is a GCS score of 9 to 13.

The rationale was primarily to identify all patients needing neurosurgical intervention, including medical intervention for high intracranial pressure (assigned a critical level with regard to patient-important outcomes). The secondary goals (assigned important, but not critical, with regard to patient-important outcomes) were identification of non-neurosurgical intracranial traumatic complications and also strong consideration of resource use with minimization of unnecessary (normal) CT scans and/or admission.

The task force decided *a priori *to make an attempt to keep the guidelines applicable to the complete patient spectrum within EDs, that is, to ensure that all adult patients with minimal, mild and moderate head injury can be managed according to the guidelines.

Certain assumptions were also made *a priori *concerning aspects of management that were deemed unnecessary for critical review. The task force all agreed that magnetic resonance imaging (MRI) would not be considered in these guidelines concerning initial management and that in-hospital observation, instead of CT, would be regarded only as a secondary management option. The use of plain skull films was addressed and rejected in the previous guidelines. Additionally, we chose not to consider later aspects of management, such as detection and treatment of post-concussion syndrome (PCS) and chronic subdural hematomas. We also agreed that all pathological findings on head CT should lead to hospital admission. Finally, we would not address the surgical or medical management of intracranial complications.

The task force was unclear concerning the selection of patients for CT scanning or discharge, following minimal, mild and moderate head injuries. We were also unclear concerning which patients, irrespective of initial CT scan results, should have hospital admission for clinical observation, a repeat CT scan, or both. Therefore, consensus was achieved to address two important clinical questions that would require systematic review of evidence and would form the basis of the updated guidelines, shown below.

Clinical question 1: 'Which adult patients with minimal, mild and moderate head injury need a head CT and which patients may be directly discharged?'.

Clinical question 2: 'Which adult patients with minimal, mild and moderate head injury need in-hospital observation and/or a repeat head CT?'.

### Search strategy

In order to address the clinical questions we performed two separate systematic reviews of the literature, in accordance to the Preferred Reporting Items for Systematic Reviews and Meta-Analyses (PRISMA) statement [22]. Both utilized broad searches of the MEDLINE and EMBASE databases, from 1985 until January 2010 and then complemented to July 2012, using prespecified Medical Subject Headings (MeSH) terms and key words depicted by the task force. MeSH terms were pretested for validity through identification of several key articles. It was deemed unlikely that studies prior to 1985 would be useful considering the wide-scale introduction of CT scanning around this period. We did not apply any other limitations to the search.

For the first clinical question, the MeSH terms and keywords were; ((head trauma) OR (brain injury) OR (head injury) OR (traumatic head injury) OR (traumatic brain injury)) AND (minimal OR mild OR minor OR moderate) AND (management OR predictors OR predictor).

For the second clinical question we used; ((head trauma) OR (brain injury) OR (head injury) OR (traumatic head injury) OR (traumatic brain injury)) AND (minimal OR mild OR minor OR moderate) AND (hospitalization OR hospitalisation OR observation OR admission OR discharge OR delayed OR ((normal OR negative OR repeat OR multiple OR serial OR follow-up) AND (CT OR CCT OR computed tomography)).

Additional papers were identified by hand-searching bibliographies of retrieved studies.

### Selection criteria and study eligibility

Titles were examined by one author (JU) and borderline titles were included. Titles that were obviously not relevant were excluded. Abstracts were examined independently by two authors (JU, BR) and the third (TI) was consulted when discrepancies arose. Selected full papers were independently reviewed by all authors (JU, TI, BR) and discrepancies were resolved through discussion.

Review articles, letters, expert opinion and editorials could be retrieved for examination of bibliographies but were excluded from the analysis. Papers reporting only children (<18 years) were excluded in both searches. In cases where essential data was missing or unclear, we made an attempt to contact corresponding authors for clarification. Studies including patients with all severities of head injury were only included if at least 50% of patients were within the GCS 9 to 15 range.

For the first clinical question, we included studies reporting patients with admission/initial GCS scores ≥9 and that included one or more predictive risk factors for the reference standards of CT findings, intracranial injury (ICI) and/or neurosurgical intervention. We decided *a priori *to only include studies where information concerning true positives (TP), false positives (FP), true negatives (TN) and false negatives (FN) could be extracted. This information would be necessary to fully appreciate the possible clinical effect and role of a risk factor, allowing consideration of other effects than the positive predictive power. Studies reporting less than 50 patients were excluded. Definitions for risk factors were defined *a priori*.

For the second clinical question, we included studies reporting patients with admission/initial GCS scores ≥9 with an initial CT scan (normal or abnormal) and contained information regarding clinical characteristics that were associated with a positive or worsening repeat CT scan, ICI and/or neurosurgical intervention within 1 week following trauma.

CT findings were defined as any traumatic finding on head CT. ICI was defined as any intracranial (isolated non-depressed cranial fractures not included) traumatic finding on CT. Also, since not all patients can be subjected to CT, absence of ICI was defined as relevant and robust clinical follow-up suggestive of normal neurological functioning (with the exception of classical PCS symptoms). The decision to consider any CT findings and ICI as separate reference standards was due to the difference in clinical importance of these measures. This approach should also stratify reference standards in a more homogenous selection compared to a combined definition. Finally, neurosurgical intervention was defined as any neurosurgical procedure for a cranial or intracranial injury within the first week following trauma. Medical treatment for elevated intracranial pressure, within the first week following trauma, was also included in this group since some patients with diffuse brain injury cannot be managed surgically.

### Data extraction and quality assessment

Data was extracted by one author (JU) and checked by another (BR). Data was entered into a predefined protocol and then inputted into Excel (Microsoft; Redmond, WA, USA). Evidentiary tables were constructed to summaries the studies. We decided to address the quality of papers in different phases, due to the nature of the studies and the phase of assessment. Firstly, all retrieved studies were independently graded by all authors in the task force (JU, TI, BR) according to the Centre of Evidence Based Medicine (CEBM) diagnosis criteria [23]. Discrepancies in grading were resolved through discussion. Quality ratings ranged from 1 (strongest evidence, for instance reports of clinical decision rules and high quality validation studies) to 5 (weakest evidence, often expert opinion). Studies receiving CEBM scores of 5 were excluded.

Studies were then graded according to the Quality Assessment of Diagnostic Accuracy Studies (QUADAS) tool [24], which was modified for the purpose of the review. This tool considers 14 criteria relevant to diagnostic studies accounting for bias (items 3 to 7, 10 to 12), variability (items 1 and 2) and reporting (items 8 and 9). Items 4 (regarding the time period between index and reference test) and 7 (regarding the independency of the reference test) were omitted with regard to the selection criteria and the previously applied CEBM criteria. Additionally, item 3 (regarding the ability of the reference test to correctly classify the target condition) was applied to CT findings, ICI and neurosurgery separately.

### Data analysis

Although extracted data regarding the first clinical question, predictors of CT findings, ICI and neurosurgery, could theoretically be summarized in a meta-analysis, the task force decided *a priori *not to perform such an analysis for the purpose of development of the guidelines, independent of heterogeneity between studies. We felt combining the data in this way could mislead the working group in the consensus process and opted to instead present uncombined data for studies including their quality assessment. We therefore calculated individual positive likelihood ratios (PLR) and negative likelihood ratios (NLR) for each risk factor with respect to the corresponding reference test (CT, ICI or neurosurgery) and the prevalence of both the reference test and the risk factor in the population. We felt that these indices would represent the most relevant clinical applications for the working group when considering the recommendations. For the second clinical question, we presented only descriptive analysis.

### Evidence summary and recommendations draft

Recommendations were formed by the task force (JU, TI, BR) based upon the evidence in accordance with the GRADE system [20,25]. This system is increasingly been used in the development of recommendations and allows consideration of aspects other than level of evidence in determining the strength of a recommendation [25]. Clinical predictors were chosen based upon the summarized evidence (see Additional file 1, Table S1). Focus was put on the more severe outcome variables (need for neurosurgery being of critical importance), but ICI and any CT findings were also considered, especially in cases where the evidence concerning neurosurgery was poor and/or inconsistent. We also considered the prevalence of the risk factors in the studied cohorts. Risk factors relatively common in a population would lead to many CT scans and these risk factors would therefore need to show high predictive abilities to be included.

The summarized quality of evidence, from studies forming the basis of a recommendation, was graded from high quality to very low quality, see Table 1. Evidence was initially considered high quality when derived from cohort studies reporting patients with diagnostic uncertainty and appropriate reference standards, as described earlier. Evidence could be downgraded due to risk of bias (selection (population indirectness), verification, observer and reporting), outcome indirectness (balance between the presumed influence on patient outcome of the test result (combination of risk factors) in relation to the complications and resource use of the test), inconsistency (large differences in prevalence of reference tests, prevalence of risk factors, PLR or NLR) or differing general results between studies), impreciseness (studies with small number of patients and few positive CT, ICI or neurosurgery events) and suspicion of publication bias (small number of studies, industry funding).

**Table 1:** Grading of Recommendations Assessment, Development and Evaluation (GRADE) system [24] for rating quality of evidence and strength of **recommendation**

Factor	Description
Evidence:	
High quality	Considerable confidence of the estimate of effect. Further research is very unlikely to change our confidence in the estimated effect.
Moderate quality	Confidence that the estimate is close to the truth. Further research is likely to have an important impact on our confidence in the estimate effect and may change the estimate.
Low quality	Limited confidence in the effect. Further research is likely to have an important impact on our confidence in the estimate effect and is likely to change the estimate.
Very low quality	Little confidence in the effect estimate. Any change of effect is uncertain.
Recommendation:	
Strong: 'We recommend...'	A strong recommendation indicates that most well informed people will make the same choice
Weak: 'We suggest...'	A weak recommendation indicates that the majority of well informed people will make the same choice but a substantial minority will not
Uncertain: 'We cannot recommend...'	No specific recommendation for or against

Factors influencing the strength of the recommendation include evidence quality, risk/benefit aspects of presumed patient-important outcomes, costs and uncertainty concerning values and preferences.

Recommendations, relating to the clinical questions, were classed as strong (we recommend...), weak (we suggest...) or uncertain (we cannot recommend...) (see Table 1). For this process, careful consideration was again made to risk/benefit aspects of patient-important outcomes (need for neurosurgery was classed as the most important) in relation to test results, including assumptions for pretest probabilities (different magnitudes of risk for a positive reference result of CT, ICI or neurosurgery) for different patients, quality of evidence, uncertainty of the preferences and values for outcomes and the use of health care resources. Therefore, it is theoretically possible to achieve a strong recommendation despite low quality evidence or vice versa.

### Recommendations and guideline development

Based upon the recommendations, a draft for the updated guidelines was constructed by the task force. Following this, a modified Delphi process was used [26], involving the working group previously described, consisting of at least two rounds of consensus. The *a priori *criteria to determine acceptance, rejection or lack of consensus are shown in Table 2. In the first round, the recommendations, including data from included studies with CEBM, QUADAS and GRADE evaluations together with a guideline draft were sent via email to the working group. Ratings, including feedback, were anonymously collected. The task force adjusted the recommendations and draft based upon these responses. Then, in conjunction with a 2-day SNC meeting in September 2012 outside Copenhagen, Denmark, results were discussed and suggestions for improvements made. Following this, the second round of Delhi was completed via email. Additional rounds would be undertaken if necessary. The task force and working group were urged to consider the GRADE aspects previously mentioned, especially health risk/benefit aspects including resource use, as well as side effects and risks (misclassification of patients), at all stages of development.

**Table 2:** *A priori *established seven-point response scale and criteria to determine acceptance, rejection or lack of consensus for recommendations and guidelines for the working group using a modified Delphi process [25]

	Level of agreement						
	
	Strongly disagree	Disagree	Moderately disagree	Neither agree or disagree	Moderately agree	Agree	Strongly agree
Score	1	2	3	4	5	6	7
Criteria	75% of respondents score ≤3 on the 7-point scale			All other situations	75% of respondents score 5≥ on the 7-point scale		
Result	Consensus against			No consensus	Consensus in favor		
Action	Reject recommendation			Indicates no consensus has been reached	Accept recommendation		

The final guidelines were evaluated, independently of the task force and working group, in the ED of Skane University Hospital, Malmo, Sweden, to judge clarity of presentation and ease of use. Simultaneously, the guidelines were evaluated by important stakeholders from specialties directly involved in the everyday management of these patients. Feedback was documented and appropriate changes were made, if necessary, but only to consensus aspects. Finally, the working group reapproved the guidelines after presentation of changes and feedback from the evaluation.

### Implementation, monitoring and future updates

Guidelines will only be successful if they are used correctly and on a wide scale. Previous experience with the 2000 Scandinavian guidelines has shown poor compliance and varying degrees of implementation success [27,28]. Implementation and monitoring strategies were discussed within the working group in order to facilitate long-term successful use of the guidelines in Scandinavia. Focus was put on overcoming barriers to application and effectively using available resources. The working group also outlined a procedure and approximate time period for updating the guidelines.

## Results

**Figure 2:**
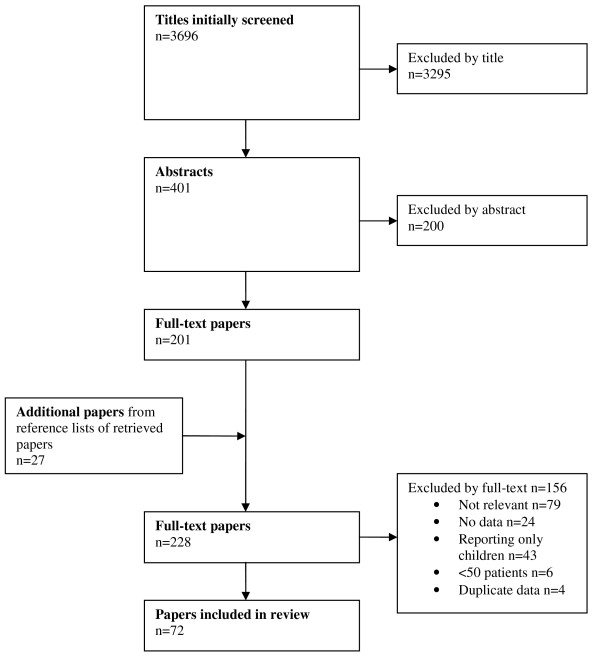
**Adapted Preferred Reporting Items for Systematic Reviews and Meta-Analyses (PRISMA) diagram showing the review process with reference to the clinical question: 'Which adult patients with minimal, mild and moderate head injury need a head CT and which patients may be directly discharged?'**.

**Figure 3:**
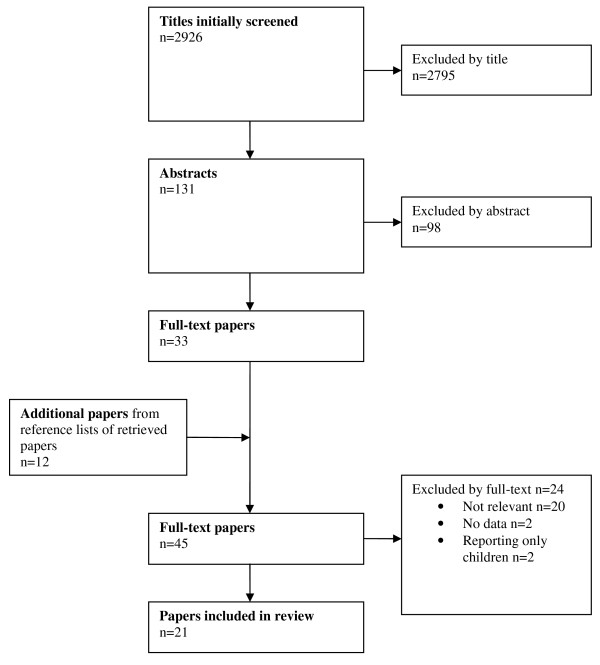
**Adapted Preferred Reporting Items for Systematic Reviews and Meta-Analyses (PRISMA) diagram showing the review process with reference to the clinical question: 'Which adult patients with minimal, mild and moderate head injury need in-hospital observation and/or a repeat head CT?'**.

The search and selection process is shown in Figures 2 and 3 for the two clinical questions.

For the first clinical question, we found 72 studies that adhered to our inclusion criteria (see Additional File 2, Table S2 for evidentiary information). These studies included 226,606 individual patients. The level of evidence according to CEBM was variable and overall judged to be moderate (see Additional file 2, Table S2). Quality assessment with the QUADAS tool (see Additional file 3, Table S3) showed substantial bias in the studies, particularly concerning the representativeness of the studied population (selection bias, criteria 1), blinding of the index test (criteria 8) and withdrawals (criteria 12). Studies scored better regarding the reporting of selection criteria (criteria 2) and most had acceptable reference standards (criteria 3), although they were often described poorly.

Clinical predictors, with according source study, PLR, NLR, reference test prevalence and risk factor prevalence are shown in Additional file 1, Table S1.

With regard to the second clinical question, we found 21 studies adhering to our inclusion criteria (see Additional file 4, Table S4 for evidentiary information and relevant results). The CEBM rating was generally low, with several studies reporting non-independent reference standards (see Additional file 4, Table S4). QUADAS assessment showed selection bias in most studies (criteria 1). Other consistent weaknesses of the studies were a lack of reference test description and blinding (see Additional file 5, Table S5 for details).

### Recommendations

Based upon the evidence, drafts for recommendations, guidelines and written discharge advice were constructed by the task force. These, with according presentation of the evidence (Additional files 1, 2, 3, 4, 5, Tables S1-S5), were reviewed by the working group using the predefined Delphi process. Following round 1 (see Table 3), discussion in the working group concerned points 4 and 7. Since point 7 regarded the overall guidelines, minor adjustments were also made to other points. Only consensus points were changed (the risk factors shunt-treated hydrocephalus and the combination of age >65 and antiplatelet medication were added, discharge advice was simplified, monitoring routines were adjusted and the graphical layout of the guidelines was improved).

**Table 3:** Results of the modified Delphi process, round 1

Delphi point	Working group member										Result	Cf/nC/Ca
			
	1	2	3	4	5	6	7	8	9	10		
1	6	6	6	6	2	6	6	5	7	7	90%	Cf
2	6	6	6	-	3	6	6	6	4	7	78%	Cf
3	7	6	7	6	3	6	6	7	1	7	80%	Cf
4	5	4	4	6	2	6	6	6	6	2	60%	nC
5	6	5	6	6	7	6	6	7	6	6	100%	Cf
6	6	6	7	4	7	6	6	6	7	7	90%	Cf
7	6	6	5	6	2	6	6	-	4	3	67%	nC
8	5	5	6	6	6	6	6	7	-	3	89%	Cf
9	5	-	5	6	6	6	6	6	-	5	100%	Cf

Delphi points 1 to 3 refer to recommendations 1 to 3 concerning clinical question 1, point 4 refers to a recommendation that was dropped due to irrelevance (see main text), points 5 and 6 refer to recommendations 1 and 2 concerning clinical question 2, point 7 refers to the guideline draft including the help sheet, point 8 refers to the written discharge advice and point 9 to the in-hospital monitoring routines.

Ca = consensus against; Cf = consensus in favor; nC = no consensus.

Following round 2 (see Table 4), consensus was achieved in favor of all recommendations, the guidelines and the discharge instructions. One recommendation, concerning clinical question 1, was removed due to the working group finding the information irrelevant, despite consensus. This recommendation was an uncertain recommendation (we cannot recommend...) for risk factors not included in the other recommendations (such as headache, intoxication, nausea and amnesia). The working group felt this recommendation was unnecessary and confusing, shifting focus from the important recommendations below.

**Table 4:** Results of the modified Delphi process, round 2

Delphi point	Working group member										Result	Cf/nC/Ca
			
	1	2	3	4	5	6	7	8	9	10		
1	7	6	3	6	7	7	6	7			88%	Cf
2	-	6	4	6	6	6	6	7			86%	Cf
3	7	6	3	6	6	6	7	7			88%	Cf
4	7	6	6	6	6	3	7	6			88%	Cf
5	7	6	7	6	7	-	6	7			100%	Cf
6	7	7	7	6	7	-	-	7			100%	Cf
7	7	6	7	6	6	7	5	7			100%	Cf
8	7	6	4	-	-	-	6	7			80%	Cf
9	7	6	6	5	7	-	6	7			100%	Cf

Two members did not reply. Delphi points 1 to 3 refer to recommendations 1 to 3 concerning clinical question 1, point 4 refers to a recommendation that was finally dropped due to irrelevance (see main text), points 5 and 6 refer to recommendations 1 and 2 concerning clinical question 2, point 7 refers to the guideline draft including the help sheet, point 8 refers to the written discharge advice and point 9 to the in-hospital monitoring routines.

Ca = consensus against; Cf = consensus in favor; nC = no consensus.

The final recommendations, based purely on evidence, are presented below.

### Clinical question 1: 'Which adult patients with minimal, mild and moderate head injury need a head CT and which patients may be directly discharged?'

(1) We recommend that adult patients after mild and moderate head injury with GCS ≤14, loss of consciousness, repeated (≥2) vomiting, anticoagulant therapy or coagulation disorders, clinical signs of depressed or basal skull fracture, post-traumatic seizures or focal neurological deficits should have a CT scan (moderate quality, strong recommendation).

The evidence was initially of high quality but was downgraded due to limitations in study design (mostly selection bias), indirectness (outcomes were rarely reported) and impreciseness (different magnitudes of predictive power of risk factors between studies). However, the strength of the recommendation was view as strong by the working group, considering the seriousness of the complication and health/economic impact of missing a patient with a neurosurgical lesion. The working group also discussed older age (≥60 years and ≥65 years) as well as antiplatelet medication as risk factors of importance, partly due to the presence of these criteria in other guidelines and decision rules. However, the predictive ability was only moderate and these individual risk factors would lead to an unacceptable CT increase and so consensus was not to include these in our recommendation.

(2) We recommend that adult patients after mild head injury with GCS 14 and no risk factors (anticoagulant therapy or coagulation disorders, post-traumatic seizures, clinical signs of depressed or basal skull fracture, focal neurological deficits), or GCS 15 with loss of consciousness or repeated (≥2) vomiting and no other risk factors, be sampled for analysis of S100B if less than 6 h have elapsed following trauma. If S100B is less than 0.10 μg/l, the patient may be discharged without a CT (moderate quality, strong recommendation).

The evidence was initially of high quality but was downgraded due to study design (mostly selection bias) and indirectness (outcomes were rarely reported). However, studies consistently show that low S100B levels can be used to select patients who do not need a CT scan and, hence, may save valuable resources. Of the few missed patients in the literature, almost all are non-neurosurgical lesions. Some studies include risk factors such as GCS 13, anticoagulation and focal neurological deficits in the inclusion criteria. The working group, however, found these risk factors to be too predictive of intracranial injury.

This recommendation may seem conflicting with recommendation 1, above. However, S100B is recommended as an option for reducing unnecessary CT scans in a subgroup of Mild head injury patients with low risk for intracranial complication and/or neurosurgical intervention.

(3) We recommend that adult patients after minimal and mild head injury with GCS 15 and without risk factors (loss of consciousness, repeated (≥2) vomiting, anticoagulation therapy or coagulation disorders, post-traumatic seizures, clinical signs of depressed or basal skull fracture, focal neurological deficits) can be discharged from the hospital without a CT scan (moderate quality, strong recommendation).

The evidence was initially of high quality but was downgraded due to limitations in study design (mostly selection bias), indirectness (outcomes were rarely reported) and impreciseness (different magnitudes of predictive power of risk factors between studies). The working group felt, however, that the large proportion of patients with head injury would fall into this category and that a CT policy in all these patients would not be health/economically viable considering the very low risk of intracranial injury, and even lower risk of neurosurgery, in this patient group. As previously discussed, older age and antiplatelet medication was again considered but rejected by the working group.

### Clinical question 2: 'Which adult patients with minimal, mild and moderate head injury need in-hospital observation and/or a repeat head CT?'

(1) We suggest that all adult patients after head injury with GCS ≤13, clinical signs of depressed or basal skull fracture, anticoagulation therapy or coagulation disorder, post-traumatic seizure or focal neurological deficit should have a CT scan and be admitted to hospital for observation, irrespective of CT findings (low quality, weak recommendation).

The evidence was sparse and also of low quality due to study limitations (selection bias) and inconsistency in findings. The working group felt that it would not be good clinical practice to discharge patients with any of these risk factors, despite the low quality of evidence.

(2) We recommend that repeat CT scans should be performed in patients with neurological and/or GCS (≥2 points) deterioration (low quality, strong recommendation).

The evidence was of moderate quality and was downgraded due to serious limitations in study design and some inconsistency. Most of the evidence indicates that routine repeat CT of these patients with or without CT findings is unnecessary in the absence of clinical deterioration, specifically deterioration of GCS >2 points and/or neurological status. A strong recommendation was chosen in spite of weak evidence due to the seriousness of the condition. Clinical aspects such as anticoagulation and persistent neurological findings were discussed but the working group could not reach consensus on a recommendation for follow-up scans in these patients.

### Guidelines

Based upon the recommendations, guidelines were constructed. The addition of shunt-treated hydrocephalus was based upon consensus in the working group with little evidence to support this. The working group discussed risk factors relating to trauma mechanism and multitrauma injuries but found these difficult to recommend, mainly due to practical issues with clinical application. We considered serious extracranial injuries (defined as Abbreviated Injury Score (AIS) >3 to any organ system, for instance large (for example, femur) fractures or serious thoracic or abdominal injuries) as a risk factor due to the probability of a higher magnitude of trauma, need for extracranial CT and the poorer prognosis of brain injury in these patients. However, we finally decided to omit this as a risk factor primarily due to the difficulty of classifying this risk factor in a busy clinical scenario. Additionally, predictive ability was generally only moderate for these risk factors. Also, loss of consciousness was expanded to suspected/confirmed loss of consciousness, as it is often difficult to confirm this finding in the clinical setting. Patients who could not clearly deny any loss of consciousness should be classed as suspected. Finally, the working group could not recommend older age or antiplatelet medication as individual risk factors due to the unacceptable CT increase such a recommendation would cause, in combination with only moderate predictive abilities. However, consensus was reached to combine these into one risk factor, namely age ≥65 years and antiplatelet medication.

Written instructions for patients being discharged were adapted from the 2000 guidelines with consideration of the National Institute of Clinical Excellence (NICE) instructions [29] and a proposal for evidence-based instructions from Fung *et al. *[30], (see Additional file 6, Figure S1). With the Scandinavian setting in mind, the discharge sheet was heavily simplified for clarity. Observation and monitoring routines for admitted patients were based on consensus in the working group. We discussed the intensity of monitoring routines in relation to the severity of the complications and burden on hospital wards and finally decided that these should be relatively frequent shortly after trauma (the first 4 h) with de-escalation over time. Reasonably, most admitted patients will arrive to a ward after at least 4 h and hence already have passed the 15-minute interval period. Also, these monitoring routines will only be used in small minority of patients as moderate and high-risk patients are relatively uncommon and other patients should preferentially have a CT.

Feedback from ED evaluation and from stakeholders resulted in minor changes to wording and general appearance of the guidelines. All stakeholders and the working group approved the final version, see Additional file 7, Figure S2.

### Implementation, monitoring and future updates

The working group decided on implementation by SNC members in their respective countries. This would be performed through a combination of written and oral presentations in national medical journals and national meetings, respectively. We discussed barriers to implementation and decided that the most important of these was probably the absence of sufficient education concerning head injury management in Scandinavia. We would attempt to further facilitate implementation by printing flyers and placards with the guidelines and to send these to Scandinavian hospitals. We would also initiate national training initiatives within the respective Scandinavian countries.

With respect to monitoring aspects, the working group decided to plan a questionnaire to Scandinavian physicians treating head injury to determine the present use of guidelines, similar to previous efforts [31]. At 1 year following implementation, a follow-up questionnaire will be sent out to establish changes in management routines. We will also initiate studies examining compliance with the guidelines, as previously established in Norway [27,28], and attempt to improve insufficient use of the guidelines depending on these results. Finally, we will initiate a prospective validation study, also comparing the performance of our guidelines with other guidelines, decision rules and, importantly, unstructured physician judgment [32].

The working group decided that an update of the guidelines would be necessary in 2015. This would include evidence updates concerning the clinical questions addressed in the present update and would further examine the observation and monitoring routines for admitted patients.

## Discussion

Since 2000, considerable evidence has emerged concerning the initial management, particularly risk factors for CT selection, of minimal, mild and moderate head injury. The work presented here is, in contrast to our previous guidelines, confined to adults but a similar effort regarding management of children is underway. Although these guidelines can theoretically be used in any setting, they were designed with the Scandinavian emergency care setting in mind. They are also designed to primarily identify patients needing neurosurgical or medical intervention, with traumatic CT findings being the secondary identification goal.

In summary, the evidence was of reasonable quality referring to the predictive ability of risk factors for complications following head injury in these patients. Unsurprisingly, many of the risk factors included here are also found in other guidelines and decision rules [6,9-11,29,33]. However, several differences can be noted. We found that the predictive power of amnesia was too low to be included. This risk factor was present in the SNC guidelines from 2000, mostly due to the difficultly in ruling out loss of consciousness in some patients. For this reason, we include suspected loss of consciousness as a risk factor.

Risk factors such as intoxication, trauma above the clavicles, nausea, vertigo and headache were not included due to poor predictive ability combined with a high prevalence of these factors in the head injury population. The working group found injury mechanisms complicated to use practically in initial management and decided not to include these as risk factors.

Older age, most often defined as ≥60 or ≥65 years, is often included in other guidelines. The predictive ability of this risk factor was only moderate and there was considerable uncertainty in the group with regard to patient important outcomes and resource use. The number of people in older age groups in industrialized countries is increasing [34] and the increased CT rate that would be associated with this risk factor was deemed unacceptable. Also, the risk factor is common in the head injury cohorts, with between 10% and 45% of patients being over 65 years of age in reported cohorts of mild [10,35-38] and moderate [39] TBI. Fabbri *et al*. recently presented results considering the combination of older age and antiplatelet agents [40]. Despite the lack of good evidence for this combination, consensus was reached to include age ≥65 years in combination with any antiplatelet agent as a risk factor. It is reasonable to expect that the combination would be more predictive of complications after head injury and result in a smaller CT rate increase when compared to the risk factors used individually. Additionally, it has been suggested that antiplatelet medication may be at least partly responsible for the higher risks for intracranial complications seen after head injury in older patients [40].

Shunt-treated hydrocephalus was added purely based on consensus, with evidence derived from expert opinion in the group. We acknowledge the poor evidence-based background to this decision but this patient group is uncommon and will not lead to a noticeable increase in CT scanning.

Evidence concerning repeat CT was reasonable but lacking concerning both written discharge advice and observation routines. These aspects were therefore based heavily upon consensus with special weight put on adaptation to the Scandinavian health care system. Since in-hospital observation consumes valuable resources, there is a need for stronger evidence examining the need and magnitude of these routines.

For the first time, a brain biomarker has been introduced into clinical practice guidelines. Using a low cut-off of 0.10 μg/l, the biomarker has shown considerable ability to predict the absence of CT pathology and neurosurgical intervention [36,41,42]. This negative prediction is welcomed since all other risk factors are of positive predictive nature. S100B allows for a safe reduction in CT scans in a subpopulation of patients with mild head injury. In order to maintain the theoretical safety and cost-saving ability, the biomarker should primarily not exhibit false negative results. Also, the biomarker should only be taken in patients that would usually receive a CT scan and the fraction of negative S100B results (below cut-off) should be as large as possible. S100B is clinically unspecific [43,44] and has a short half-life [45]. Therefore, patients with extracranial injuries and those seeking care more than 6 h after trauma are not good candidates for S100B sampling due to a risk of false positives and negatives, respectively. Some patients have risk factors with higher predictive abilities and also factors that would usually warrant admission irrespective of CT findings. This group is therefore also not suitable for S100B sampling. Despite the relatively good evidence for S100B in this setting, biomarkers have historically had different effects in actual management and the clinical impact and health economic implications may alter future recommendations. Based upon the current evidence and clinical setting, however, this biomarker should safely reduce resource use if used correctly since low levels are very uncommon in patients needing neurosurgical intervention in this setting.

There are limitations to the process outlined in this paper. Although the recommendations are based upon evidence, there were elements of consensus input to the final guidelines. This is inevitable when dealing with these injuries and we attempted to minimize the negative effects of this through our stringent and extensive methodology using the best available tools. Particularly, the GRADE system [20] allows consideration of other important aspects other than the level of evidence in recommendations. The derivation and validation of predictive risk factors as performed by other authors [6,10,18] would hardly be feasible in Scandinavia and would only account for one aspect of the management guidelines. Our methodology was judged as the most feasible considering the target population. However, external clinical validation of our guidelines is welcomed and would naturally support successful implementation.

Finally, these guidelines are, by definition, guidelines and should be utilized accordingly. They are primarily designed as evidence and consensus-based guidance for physicians who are not experts in the field. Physicians who have considerable experience with these patients should naturally be allowed to defer from the guidelines according to clinical judgment.

## Conclusions

We present guidelines for initial management of adults with minimal, mild and moderate head injury based upon a thorough evidence and consensus-based methodology. The guidelines are primarily designed to detect complications after head injury needing either neurosurgical or medical intervention. They can be applied to all adult patients and include aspects such as CT and admission selection, repeat CT selection, monitoring routines and discharge aspects. However, we suggest external validation before they are widely implemented. Furthermore, areas with poor evidence, such as clinical monitoring routines for patients following head injury, should be addressed in future studies.

## Competing interests

JU and BR are organizers of the international BMBD conference (http://www.bmbd.org) concerning neurological biomarkers in brain injury. Some SNC meetings have been sponsored by Roche Diagnostics Scandinavia AB. These sponsors have had no involvement or influence on any aspects of the SNC work. JU and BR have been invited to speak at educational meetings arranged by Roche Diagnostics Scandinavia AB but Roche have always remained strictly independent from the scientific program and content and these took place before the present work was initiated. JU, TI and BR have in previous research received compensation for travel expenses and lab analysis from Roche AB, Diasorin AB and Sangtec Medical AB. None of these had any involvement or influence on any scientific aspects.

## Authors' contributions

This work was initiated and designed by JU with input from the SNC committee. JU, TI and BR all participated in the review process and recommendation/guideline draft, see text for details. For the Delphi process, the entire SNC working group (see below), including JU, TI and BR, was involved. Clinical stakeholders were involved in later processes of guideline approval, see main text. All main authors approved the final manuscript.

## Pre-publication history

The pre-publication history for this paper can be accessed here:

http://www.biomedcentral.com/1741-7015/11/50/prepub

## Supplementary Material

Additional file 1 **Table S1**. Predictive risk factors with according studies derived from the clinical question: 'Which adult patients with minimal, mild and moderate head injury need a head CT and which patients may be directly discharged?' showing corresponding positive likelihood ratio (PLR), negative likelihood ratio (NLR), prevalence for the reference test (CT findings (CT)), intracranial injury (ICI) and neurosurgery (NS)) and the risk factor prevalence. CT = computed tomography. Click here for file

Additional file 2 **Table S2**. Evidentiary table of studies with reference to the clinical question: 'Which adult patients with minimal, mild and moderate head injury need a head CT and which patients may be directly discharged?'. CT = computed tomography. Click here for file

Additional file 3 **Table S3**. Modified Quality Assessment of Diagnostic Accuracy Studies (QUADAS) grading of studies referring to the clinical question: 'Which adult patients with minimal, mild and moderate head injury need a head CT and which patients may be directly discharged?'. CT = computed tomography. Click here for file

Additional file 4 **Table S4**. Evidentiary table of studies with reference to the clinical question: 'Which adult patients with minimal, mild and moderate head injury need in-hospital observation and/or a repeat head CT?'. CT = computed tomography. Click here for file

Additional file 5 **Table S5**. Modified Quality Assessment of Diagnostic Accuracy Studies (QUADAS) grading of studies referring to the clinical question: 'Which adult patients with minimal, mild and moderate head injury need in-hospital observation and/or a repeat head CT?'. CT = computed tomography. Click here for file

Additional file 6 **Figure S1**. Discharge advice for adults following minimal, mild and moderate head injury. Click here for file

Additional file 7 **Figure S2**. Final management guidelines for adults following minimal, mild and moderate head injury (including help sheet). Click here for file
