# Supplementary material for: Scandinavian guidelines for initial management of minimal, mild and moderate head injuries in adults: an evidence and consensus-based update
Source: BMC Med. 2013 Feb 25;11:50. doi: 10.1186/1741-7015-11-50 (PMC3621842; doi:10.1186/1741-7015-11-50)
Supplement: Additional file 1 — Table S1. Predictive risk factors with according studies derived from the clinical question: 'Which adult patients with minimal, mild and moderate head injury need a head CT and which patients may be directly discharged?' showing corresponding positive likelihood ratio (PLR), negative likelihood ratio (NLR), prevalence for the reference test (CT findings (CT)), intracranial injury (ICI) and neurosurgery (NS)) and the risk factor prevalence. CT = computed tomography. [file 1741-7015-11-50-S1.DOC]

Additional File 1: Table S1. a)-ai). Predictive risk factors with according studies derived from the clinical question: “Which adult patients with minimal, mild and moderate head injury need a head CT and which patients may be directly discharged?” showing corresponding Positive Likelihood Ratio (PLR), Negative Likelihood Ratio (NLR), prevalence for the reference test (CT findings (CT), intracranial injury (ICI) and neurosurgery (NS)) and the risk factor prevalence. GCS = Glasgow Coma Scale. Prev=Prevalence.

| **a) Vomiting** |  | | | | | | | | | | |
| --- | --- | --- | --- | --- | --- | --- | --- | --- | --- | --- | --- |
| **Study** | **Year** | **Prev risk factor** | **PLR CT** | **NLR CT** | **Prev CT** | **PLR ICI** | **NLR ICI** | **Prev ICI** | **PLR NS** | **NLR NS** | **Prev NS** |
| Haydel MJ et al | 2000 | 9.0% |  |  |  | 1.3 | 1.0 | 6.9% |  |  |  |
| Smits M et al | 2007 | 10.1% |  |  |  | 2.3 | 0.9 | 2.4% |  |  |  |
| Abdul Latip LS et al | 2003 | 42.6% |  |  |  | 1.8 | 0.7 | 53.2% |  |  |  |
| Fabbri A et al | 2010 | 2.6% |  |  |  | 12.9 | 0.8 | 6.2% |  |  |  |
| Holmes JF et al | 1997 | 3.8% |  |  |  | 6.5 | 0.9 | 13.3% | 7.2 | 0.8 | 1.5% |
| Mack LR et al | 2003 | 8.3% |  |  |  | 2.3 | 0.9 | 14.3% |  |  |  |
| Saboori M et al | 2007 | 5.7% |  |  |  | 3.6 | 0.9 | 6.7% | 14.1 | 0.3 | 0.6% |
| Stiell IG et al | 2001 | 9.6% |  |  |  | 3.9 | 0.8 | 8.1% |  |  |  |
| Turedi S et al | 2008 | 26.3% | 3.3 | 0.5 | 19.6% |  |  |  | 1.0 |  | 3.2% |
| Jeret JS et al | 1992 | 7.4% |  |  |  | 1.7 | 0.9 | 9.7% | 6.9 | 0.5 | 0.3% |
| Falimirski ME et al | 2003 | 5.7% | 0.6 | 1.1 | 12.1% |  |  |  |  |  |  |
| Ibanez J et al | 2004 | 8.0% |  |  |  | 3.4 | 0.8 | 7.5% |  |  |  |
| Hsiang JNK et al | 1997 | 25.3% | 1.0 | 1.0 | 22.0% |  |  |  |  |  |  |
| Miller EC et al | 1997 | 5.7% | 3.0 | 0.9 | 6.4% |  |  |  | 0.0 | 1.1 | 0.2% |
| Mower WR et al | 2005 | 5.8% |  |  |  | 1.9 | 0.9 | 6.7% |  |  |  |
| Duus BR et al | 1993 | 27.3% |  |  |  | 2.5 | 0.5 | 0.5% |  |  |  |
| Schynoll W et al | 1993 | 29.5% |  |  |  | 0.8 | 1.1 | 12.3% |  |  |  |
| Abdul Rahman YS et al | 2010 | 38.8% | 0.9 | 1.0 | 40.8% |  |  |  |  |  |  |
| Ando S et al | 1992 | 26.9% |  |  |  | 1.9 | 0.7 | 11.8% |  |  |  |
| Lee ST et al | 1995 | 4.6% |  |  |  | 10.6 | 0.6 | 1.5% |  |  |  |
| Stiell IG et al | 2005 | 10.0% |  |  |  | 2.2 | 0.9 | 5.1% |  |  |  |
| Mussack 2002 | 2002 | 10.1% | 2.5 | 0.9 | 13.7% |  |  |  |  |  |  |
| Kotlyar S et al | 2010 | 4.3% | 9.8 | 0.7 | 6.4% |  |  |  |  |  |  |
| Zongo et al | 2012 | 5.3% | 1.8 | 1.0 | 7.1% |  |  |  |  |  |  |
| Ono K et al | 2007 | 7.5% | 4.3 | 0.8 | 4.7% |  |  |  |  |  |  |

| **b) Repeated (>1) vomiting** |  | | | | | | | | | | |
| --- | --- | --- | --- | --- | --- | --- | --- | --- | --- | --- | --- |
| **Study** | **Year** | **Prev risk factor** | **PLR CT** | **NLR CT** | **Prev CT** | **PLR ICI** | **NLR ICI** | **Prev ICI** | **PLR NS** | **NLR NS** | **Prev NS** |
| Stiell IG et al | 2001 | 9.6% |  |  |  | 3.9 | 0.8 | 8.1% |  |  |  |
| Mower WR et al | 2005 | 5.8% |  |  |  | 1.9 | 0.9 | 6.7% |  |  |  |
| Stiell IG et al | 2005 | 14.9% |  |  |  | 3.7 | 0.6 | 7.9% |  |  |  |
| Barrow A et al | 2012 | 12.9% | 2.2 | 0.8 | 2.2% |  |  |  |  |  |  |

| **c) Nausea** |  | | | | | | | | | | |
| --- | --- | --- | --- | --- | --- | --- | --- | --- | --- | --- | --- |
| **Study** | **Year** | **Prev risk factor** | **PLR CT** | **NLR CT** | **Prev CT** | **PLR ICI** | **NLR ICI** | **Prev ICI** | **PLR NS** | **NLR NS** | **Prev NS** |
| Holmes JF et al | 1997 | 8.0% |  |  |  | 2.1 | 0.9 | 13.3% | 4.8 | 0.6 | 1.5% |
| Borczuk P et al | 1995 | 12.5% | 1.9 | 0.9 | 8.2% |  |  |  |  |  |  |
| Jeret JS et al | 1992 | 14.8% |  |  |  | 1.4 | 0.9 | 9.7% | 3.4 | 0.6 | 0.3% |
| Ibanez J et al | 2004 | 16.3% |  |  |  | 1.9 | 0.8 | 7.5% |  |  |  |
| Miller EC et al | 1997 | 10.2% | 2.1 | 0.9 | 6.4% |  |  |  | 2.0 | 0.9 | 0.2% |
| Mussack 2002 | 2002 | 21.6% | 1.0 | 1.0 | 13.7% |  |  |  |  |  |  |
| Kotlyar S et al | 2010 | 13.9% | 2.5 | 0.8 | 6.4% |  |  |  |  |  |  |

| **d) Headache** |  | | | | | | | | | | |
| --- | --- | --- | --- | --- | --- | --- | --- | --- | --- | --- | --- |
| **Study** | **Year** | **Prev risk factor** | **PLR CT** | **NLR CT** | **Prev CT** | **PLR ICI** | **NLR ICI** | **Prev ICI** | **PLR NS** | **NLR NS** | **Prev NS** |
| Haydel MJ et al | 2000 | 23.7% |  |  |  | 1.5 | 0.9 | 6.9% |  |  |  |
| Smits M et al | 2007 | 56.0% |  |  |  | 1.2 | 0.8 | 7.6% |  |  |  |
| Fabbri A et al | 2010 | 13.4% |  |  |  | 1.4 | 0.9 | 6.2% |  |  |  |
| Holmes JF et al | 1997 | 27.3% |  |  |  | 1.9 | 0.7 | 13.3% | 0.9 | 1.0 | 1.5% |
| Borczuk P et al | 1995 | 39.8% | 1.3 | 0.8 | 8.2% |  |  |  |  |  |  |
| Mack LR et al | 2003 | 21.8% |  |  |  | 1.6 | 0.9 | 14.3% |  |  |  |
| Saboori M et al | 2007 | 13.8% |  |  |  | 0.3 | 1.1 | 6.7% | 3.7 | 0.6 | 0.6% |
| Stiell IG et al | 2001 | 58.4% |  |  |  | 1.0 | 0.9 | 7.1% |  |  |  |
| Turedi S et al | 2008 | 4.2% | 1.8 | 1.0 | 19.6% |  |  |  |  |  | 0.0% |
| Mikhail MG et al | 1992 | 56.3% |  |  |  | 1.6 | 0.3 | 7.1% |  |  | 100.0% |
| Jeret JS et al | 1992 | 22.8% |  |  |  | 0.6 | 1.1 | 9.7% | 1.4 | 0.8 | 0.2% |
| Falimirski ME et al | 2003 | 15.7% | 1.5 | 0.9 | 12.1% |  |  |  |  |  |  |
| Ibanez J et al | 2004 | 55.6% |  |  |  | 1.3 | 0.7 | 7.5% |  |  |  |
| Miller EC et al | 1996 | 35.0% | 1.7 | 0.7 | 6.1% |  |  |  |  |  |  |
| Mower WR et al | 2005 | 16.6% |  |  |  | 0.7 | 1.1 | 6.7% |  |  |  |
| Schynoll W et al | 1993 | 70.2% |  |  |  | 0.6 | 2.2 | 9.6% |  |  |  |
| Abdul Rahman YS et al | 2010 | 61.2% | 1.0 | 1.1 | 40.8% |  |  |  |  |  |  |
| Lee ST et al | 1995 | 13.7% |  |  |  | 4.4 | 0.5 | 1.4% |  |  |  |
| Stiell IG et al | 2005 | 27.2% |  |  |  | 1.4 | 0.9 | 4.1% |  |  |  |
| Kotlyar S et al | 2010 | 42.8% | 2.0 | 0.3 | 6.4% |  |  |  |  |  |  |
| Zongo et al | 2012 | 17.5% | 1.3 | 0.9 | 7.1% |  |  |  |  |  |  |
| Barrow A et al | 2012 | 40.0% | 1.4 | 0.8 | 2.2% |  |  |  |  |  |  |
| Ono K et al | 2007 | 15.3% | 2.7 | 0.7 | 4.7% |  |  |  |  |  |  |

| **e) Progressive or severe headache** |  | | | | | | | | | | |
| --- | --- | --- | --- | --- | --- | --- | --- | --- | --- | --- | --- |
| **Study** | **Year** | **Prev risk factor** | **PLR CT** | **NLR CT** | **Prev CT** | **PLR ICI** | **NLR ICI** | **Prev ICI** | **PLR NS** | **NLR NS** | **Prev NS** |
| Turedi S et al | 2008 | 4.2% | 1.8 | 1.0 | 19.6% |  |  |  |  |  | 0.0% |
| Ibanez J et al | 2004 | 6.4% |  |  |  | 2.7 | 0.9 | 7.5% |  |  |  |
| Miller EC et al | 1997 | 32.3% | 1.8 | 0.7 | 6.4% |  |  |  | 1.2 | 0.9 | 0.2% |
| Mower WR et al | 2005 | 16.6% |  |  |  | 0.7 | 1.1 | 6.7% |  |  |  |

| **f) Dizziness** |  | | | | | | | | | | |
| --- | --- | --- | --- | --- | --- | --- | --- | --- | --- | --- | --- |
| **Study** | **Year** | **Prev risk factor** | **PLR CT** | **NLR CT** | **Prev CT** | **PLR ICI** | **NLR ICI** | **Prev ICI** | **PLR NS** | **NLR NS** | **Prev NS** |
| Mack LR et al | 2003 | 18.8% |  |  |  | 0.5 | 1.1 | 14.3% |  |  |  |
| Jeret JS et al | 1992 | 22.8% |  |  |  | 0.6 | 1.1 | 9.7% | 1.4 | 0.8 | 0.2% |
| Ibanez J et al | 2004 | 26.9% |  |  |  | 0.8 | 1.1 | 7.5% |  |  |  |
| Mussack 2002 | 2002 | 23.7% | 0.4 | 1.2 | 13.7% |  |  |  |  |  |  |
| Kotlyar S et al | 2010 | 13.0% | 0.7 | 1.0 | 6.4% |  |  |  |  |  |  |

| **g) Drowsiness** |  | | | | | | | | | | |
| --- | --- | --- | --- | --- | --- | --- | --- | --- | --- | --- | --- |
| **Study** | **Year** | **Prev risk factor** | **PLR CT** | **NLR CT** | **Prev CT** | **PLR ICI** | **NLR ICI** | **Prev ICI** | **PLR NS** | **NLR NS** | **Prev NS** |
| Falimirski ME et al | 2003 | 7.6% | 1.7 | 0.9 | 12.1% |  |  |  |  |  |  |
| Schynoll W et al | 1993 | 34.5% |  |  |  | 1.2 | 0.9 | 10.2% |  |  |  |
| Lee ST et al | 1995 | 1.3% |  |  |  |  | 0.2 | 1.6% |  |  |  |
| Barrow A et al | 2012 | 18.7% | 1.0 | 1.0 | 2.2% |  |  |  |  |  |  |

| **h) Seizures** |  | | | | | | | | | | |
| --- | --- | --- | --- | --- | --- | --- | --- | --- | --- | --- | --- |
| **Study** | **Year** | **Prev risk factor** | **PLR CT** | **NLR CT** | **Prev CT** | **PLR ICI** | **NLR ICI** | **Prev ICI** | **PLR NS** | **NLR NS** | **Prev NS** |
| Haydel MJ et al | 2000 | 4.6% |  |  |  | 2.7 | 0.9 | 6.9% |  |  |  |
| Smits M et al | 2007 | 0.7% |  |  |  | 3.4 | 1.0 | 6.6% |  |  |  |
| Fabbri A et al | 2010 | 1.8% |  |  |  | 6.3 | 0.9 | 6.2% |  |  |  |
| Saboori M et al | 2007 | 0.3% |  |  |  | 0.0 | 1.0 | 6.7% | 0.0 | 1.0 | 0.6% |
| Stiell IG et al | 2001 | 0.5% |  |  |  | 2.0 | 1.0 | 14.3% |  |  |  |
| Turedi S et al | 2008 | 0.4% |  | 1.0 | 19.6% |  |  |  |  |  | 0.0% |
| Falimirski ME et al | 2003 | 0.9% | 0.1 | 1.2 | 12.1% |  |  |  |  |  |  |
| Ibanez J et al | 2004 | 0.6% |  |  |  | 2.0 | 1.0 | 7.5% |  |  |  |
| Mower WR et al | 2005 | 3.9% |  |  |  | 1.2 | 1.0 | 6.7% |  |  |  |
| Schynoll W et al | 1993 | 3.8% |  |  |  | 0.8 | 1.0 | 12.1% |  |  |  |
| Abdul Rahman YS et al | 2010 | 3.3% | 5.8 | 0.9 | 40.8% |  |  |  |  |  |  |
| Stiell IG et al | 2005 | 2.1% |  |  |  | 2.1 | 1.0 | 5.5% |  |  |  |
| Kotlyar S et al | 2010 | 0.9% | 7.4 | 1.0 | 6.4% |  |  |  |  |  |  |
| Zongo et al | 2012 | 1.7% | 1.1 | 1.0 | 7.1% |  |  |  |  |  |  |
| Barrow A et al | 2012 | 0.6% | 0.0 | 1.0 | 2.2% |  |  |  |  |  |  |

| **i) Amnesia** |  | | | | | | | | | | |
| --- | --- | --- | --- | --- | --- | --- | --- | --- | --- | --- | --- |
| **Study** | **Year** | **Prev risk factor** | **PLR CT** | **NLR CT** | **Prev CT** | **PLR ICI** | **NLR ICI** | **Prev ICI** | **PLR NS** | **NLR NS** | **Prev NS** |
| Haydel MJ et al | 2000 | 1.7% |  |  |  | 16.8 | 0.9 | 6.9% |  |  |  |
| Smits M et al | 2007 | 14.7% |  |  |  | 2.2 | 0.8 | 7.2% |  |  |  |
| Abdul Latip LS et al | 2003 | 16.0% |  |  |  | 0.2 | 1.3 | 53.2% |  |  |  |
| Fabbri A et al | 2010 | 40.8% |  |  |  | 1.3 | 0.8 | 6.2% |  |  |  |
| Borczuk P et al | 1995 | 19.3% | 1.7 | 0.8 | 8.2% |  |  |  |  |  |  |
| Mack LR et al | 2003 | 19.5% |  |  |  | 0.8 | 1.1 | 14.3% |  |  |  |
| Saboori M et al | 2007 | 6.5% |  |  |  | 1.4 | 1.0 | 6.7% | 3.9 | 0.8 | 0.6% |
| Stiell IG et al | 2001 | 87.2% |  |  |  | 1.1 | 0.5 | 8.1% |  |  |  |
| Turedi S et al | 2008 | 12.1% | 1.8 | 0.9 | 19.6% |  |  |  | 1.0 |  | 3.4% |
| Ibanez J et al | 2004 | 9.9% |  |  |  | 2.4 | 0.9 | 7.5% |  |  |  |
| Miller EC et al | 1996 | 38.8% | 0.9 | 1.1 | 6.1% |  |  |  |  |  |  |
| Duus BR et al | 1993 | 46.6% |  |  |  | 2.2 | 0.0 | 0.5% |  |  |  |
| Schynoll W et al | 1993 | 33.3% |  |  |  | 1.6 | 0.7 | 9.2% |  |  |  |
| Abdul Rahman YS et al | 2010 | 25.7% | 1.2 | 0.9 | 40.8% |  |  |  |  |  |  |
| Stiell IG et al | 2005 | 25.4% |  |  |  | 2.4 | 0.6 | 7.0% |  |  |  |
| Mussack 2002 | 2002 | 59.7% | 1.1 | 0.9 | 13.7% |  |  |  |  |  |  |
| Kotlyar S et al | 2010 | 30.6% | 2.2 | 0.5 | 6.4% |  |  |  |  |  |  |
| Zongo et al | 2012 | 35.4% | 1.6 | 0.7 | 7.1% |  |  |  |  |  |  |
| Barrow A et al | 2012 | 4.2% | 0.0 | 1.0 | 2.2% |  |  |  |  |  |  |

| **j) Loss of consciousness** |  | | | | | | | | | | |
| --- | --- | --- | --- | --- | --- | --- | --- | --- | --- | --- | --- |
| **Study** | **Year** | **Prev risk factor** | **PLR CT** | **NLR CT** | **Prev CT** | **PLR ICI** | **NLR ICI** | **Prev ICI** | **PLR NS** | **NLR NS** | **Prev NS** |
| Smits M et al | 2007 | 60.4% |  |  |  | 1.3 | 0.6 | 3.4% |  |  |  |
| Fabbri A et al | 2010 | 29.7% |  |  |  | 2.6 | 0.4 | 6.2% |  |  |  |
| Borczuk P et al | 1995 | 64.4% | 1.2 | 0.6 | 8.2% |  |  |  |  |  |  |
| Mack LR et al | 2003 | 29.3% |  |  |  | 1.5 | 0.8 | 14.3% |  |  |  |
| Saboori M et al | 2007 | 11.4% |  |  |  | 0.7 | 1.0 | 6.7% | 2.2 | 0.8 | 0.6% |
| Stiell IG et al | 2001 | 46.0% |  |  |  | 1.1 | 0.9 | 8.1% |  |  |  |
| Turedi S et al | 2008 | 7.1% | 2.9 | 0.9 | 19.6% |  |  |  |  |  | 0.0% |
| Mikhail GM et al | 1992 | 22.3% |  |  |  | 2.5 | 0.6 | 7.1% |  |  |  |
| Moran SG et al | 1994 | 46.5% | 2.3 | 0.0 | 4.0% |  |  |  |  |  |  |
| Ibanez J et al | 2004 | 33.1% |  |  |  | 2.5 | 0.4 | 7.5% |  |  |  |
| Hung CC et al | 1996 | 41.4% |  |  |  | 1.7 | 0.6 | 12.2% |  |  |  |
| Miller EC et al | 1996 | 61.2% | 1.1 | 0.9 | 6.1% |  |  |  |  |  |  |
| Mower WR et al | 2005 | 48.4% |  |  |  | 1.3 | 0.7 | 6.7% |  |  |  |
| Livingston DH et al | 1991a | 82.0% |  |  |  | 1.0 | 1.1 | 13.5% |  |  |  |
| Nelson JB et al | 1992 | 76.1% | 1.2 | 0.5 | 15.6% |  |  |  |  |  |  |
| Schynoll W et al | 1993 | 35.2% |  |  |  | 1.5 | 0.7 | 12.1% |  |  |  |
| Abdul Rahman YS et al | 2010 | 44.7% | 0.6 | 1.5 | 40.8% |  |  |  |  |  |  |
| Brewer ES et al | 2010 | 26.5% |  |  |  | 2.3 | 0.7 | 24.2% |  |  |  |
| Stiell IG et al | 2005 | 34.2% |  |  |  | 1.6 | 0.7 | 6.1% |  |  |  |
| Mussack 2002 | 2002 | 41.7% | 1.3 | 0.8 | 13.7% |  |  |  |  |  |  |
| Kotlyar S et al | 2010 | 36.1% | 1.6 | 0.7 | 6.4% |  |  |  |  |  |  |
| Zongo D et al | 2012 | 40.2% | 1.4 | 0.7 | 7.1% |  |  |  |  |  |  |
| Barrow A et al | 2012 | 22.7% | 3.4 | 0.3 | 2.2% |  |  |  |  |  |  |
| Ono K et al | 2007 | 29.0% | 2.6 | 0.4 | 4.7% |  |  |  |  |  |  |

| **k) Loss of consciousness > 5 min** |  | | | | | | | | | | |
| --- | --- | --- | --- | --- | --- | --- | --- | --- | --- | --- | --- |
| **Study** | **Year** | **Prev risk factor** | **PLR CT** | **NLR CT** | **Prev CT** | **PLR ICI** | **NLR ICI** | **Prev ICI** | **PLR NS** | **NLR NS** | **Prev NS** |
| Stiell IG et al | 2001 | 10.2% |  |  |  | 1.7 | 0.9 | 8.1% |  |  |  |
| Miller EC et al | 1996 | 5.9% | 1.4 | 1.0 | 6.1% |  |  |  |  |  |  |
| Mower WR et al | 2005 | 8.1% |  |  |  | 4.4 | 0.8 | 6.7% |  |  |  |
| Schynoll W et al | 1993 | 7.6% |  |  |  | 7.3 | 0.7 | 12.1% |  |  |  |

| **l) Contusion** |  | | | | | | | | | | |
| --- | --- | --- | --- | --- | --- | --- | --- | --- | --- | --- | --- |
| **Study** | **Year** | **Prev risk factor** | **PLR CT** | **NLR CT** | **Prev CT** | **PLR ICI** | **NLR ICI** | **Prev ICI** | **PLR NS** | **NLR NS** | **Prev NS** |
| Smits M et al | 2007 | 38.7% |  |  |  | 1.6 | 0.7 | 11.8% |  |  |  |
| Abdul Latip LS et al | 2003 | 72.3% |  |  |  | 1.4 | 0.4 | 53.2% | 1.4 | 0.2 | 20.2% |
| Borczuk P et al | 1995 | 43.6% | 1.8 | 0.5 | 8.2% |  |  |  |  |  |  |
| Mack LR et al | 2003 | 47.4% |  |  |  | 1.0 | 1.0 | 14.3% |  |  |  |
| Miller EC et al | 1996 | 70.1% | 1.3 | 0.3 | 6.1% |  |  |  |  |  |  |
| Mower WR et al | 2005 | 32.6% |  |  |  | 1.5 | 0.8 | 6.7% |  |  |  |
| Kotlyar S et al | 2010 | 39.3% | 1.6 | 0.7 | 6.4% |  |  |  |  |  |  |
| Ono K et al | 2007 | 71.5% | 1.0 | 0.9 | 4.7% |  |  |  |  |  |  |

| **m) Signs of open or depressed skull fracture** |  | | | | | | | | | | |
| --- | --- | --- | --- | --- | --- | --- | --- | --- | --- | --- | --- |
| **Study** | **Year** | **Prev risk factor** | **PLR CT** | **NLR CT** | **Prev CT** | **PLR ICI** | **NLR ICI** | **Prev ICI** | **PLR NS** | **NLR NS** | **Prev NS** |
| Borczuk P et al | 1995 | 0.6% | 78.2 | 0.9 | 8.2% |  |  |  |  |  |  |
| Stiell IG et al | 2001 | 3.3% |  |  |  | 4.6 | 0.9 | 8.1% |  |  |  |
| Miller EC et al | 1997 | 0.1% |  | 1.0 | 6.4% |  |  |  |  | 0.4 | 0.2% |
| Stiell IG et al | 2005 | 3.9% |  |  |  | 4.6 | 0.9 | 9.0% |  |  |  |
| Kotlyar S et al | 2010 | 1.4% | 9.8 | 0.9 | 6.4% |  |  |  |  |  |  |
| Barrow A et al | 2012 | 0.2% |  | 0.9 | 2.2% |  |  |  |  |  |  |

| **n) Signs of basal skull fracture** |  | | | | | | | | | | |
| --- | --- | --- | --- | --- | --- | --- | --- | --- | --- | --- | --- |
| **Study** | **Year** | **Prev risk factor** | **PLR CT** | **NLR CT** | **Prev CT** | **PLR ICI** | **NLR ICI** | **Prev ICI** | **PLR NS** | **NLR NS** | **Prev NS** |
| Smits M et al | 2007 | 2.3% |  |  |  | 11.9 | 0.9 | 7.5% |  |  |  |
| Fabbri A et al | 2010 | 2.6% |  |  |  | 50.4 | 0.7 | 6.2% |  |  |  |
| Borczuk P et al | 1995 | 2.8% | 12.3 | 0.8 | 8.2% |  |  |  |  |  |  |
| Stiell IG et al | 2001 | 6.7% |  |  |  | 6.5 | 0.7 | 8.1% |  |  |  |
| Turedi S et al | 2008 | 16.7% | 3.7 | 0.7 | 19.6% |  |  |  | 1.0 |  | 5.0% |
| Mikhail GM et al | 1992 | 2.7% |  |  |  | 26.0 | 0.8 | 7.1% | 0.0 | 1.0 | 2.7% |
| Jeret JS et al | 1992 | 3.5% |  |  |  | 4.5 | 0.9 | 9.4% | 0.0 | 1.0 | 0.3% |
| Ibanez J et al | 2004 | 3.4% |  |  |  | 9.3 | 0.8 | 7.5% |  |  |  |
| Mower WR et al | 2005 | 4.2% |  |  |  | 7.3 | 0.8 | 6.7% |  |  |  |
| Schynoll W et al | 1993 | 8.0% |  |  |  | 3.6 | 0.8 | 12.1% |  |  |  |
| Stiell IG et al | 2005 | 6.0% |  |  |  | 8.1 | 0.7 | 8.8% |  |  |  |
| Kotlyar S et al | 2010 | 1.7% | 14.7 | 0.9 | 6.4% |  |  |  |  |  |  |
| Barrow A et al | 2012 | 0.2% | 0.0 | 1.0 | 2.2% |  |  |  |  |  |  |

| **o) Altered mental status** |  | | | | | | | | | | |
| --- | --- | --- | --- | --- | --- | --- | --- | --- | --- | --- | --- |
| **Study** | **Year** | **Prev risk factor** | **PLR CT** | **NLR CT** | **Prev CT** | **PLR ICI** | **NLR ICI** | **Prev ICI** | **PLR NS** | **NLR NS** | **Prev NS** |
| Borczuk P et al | 1995 | 23.9% | 2.0 | 0.7 | 8.2% |  |  |  |  |  |  |
| Mack LR et al | 2003 | 9.8% |  |  |  | 3.8 | 0.8 | 14.3% |  |  |  |
| Saboori M et al | 2007 | 13.2% |  |  |  | 0.8 | 1.0 | 6.7% |  |  |  |
| Falimirski ME et al | 2003 | 11.8% | 2.4 | 0.8 | 12.1% |  |  |  |  |  |  |
| Mower WR et al | 2005 | 27.9% |  |  |  | 2.5 | 0.5 | 6.7% |  |  |  |
| Duus BR et al | 1993 | 13.2% |  |  |  | 6.9 | 0.1 | 0.5% |  |  |  |
| Schynoll W et al | 1993 | 34.7% |  |  |  | 1.7 | 0.7 | 9.2% |  |  |  |
| Zongo et al | 2012 | 14.7% | 1.5 | 0.9 | 7.1% |  |  |  |  |  |  |

| **p) Abnormal behaviour** |  | | | | | | | | | | |
| --- | --- | --- | --- | --- | --- | --- | --- | --- | --- | --- | --- |
| **Study** | **Year** | **Prev risk factor** | **PLR CT** | **NLR CT** | **Prev CT** | **PLR ICI** | **NLR ICI** | **Prev ICI** | **PLR NS** | **NLR NS** | **Prev NS** |
| Mower WR et al | 2005 | 22.5% |  |  |  | 2.0 | 0.7 | 6.7% |  |  |  |
| Duus BR et al | 1993 | 1.0% |  |  |  | 41.5 | 0.7 | 0.5% |  |  |  |

| **q) Focal neurological deficit** |  | | | | | | | | | | |
| --- | --- | --- | --- | --- | --- | --- | --- | --- | --- | --- | --- |
| **Study** | **Year** | **Prev risk factor** | **PLR CT** | **NLR CT** | **Prev CT** | **PLR ICI** | **NLR ICI** | **Prev ICI** | **PLR NS** | **NLR NS** | **Prev NS** |
| Smits M et al | 2007 | 9.9% |  |  |  | 1.9 | 0.9 | 9.9% |  |  |  |
| Abdul Latip LS et al | 2003 | 2.1% |  |  |  | na | 1.0 | 53.2% |  | 0.9 | 20.2% |
| Fabbri A et al | 2010 | 3.8% |  |  |  | 150.1 | 0.5 | 6.2% |  |  |  |
| Livingston DH et al | 2000 | 32.4% |  |  |  | 1.3 | 0.9 | 15.6% |  |  |  |
| Borczuk P et al | 1995 | 13.3% | 0.2 | 1.1 | 8.2% |  |  |  |  |  |  |
| Saboori M et al | 2007 | 0.1% |  |  |  |  | 1.0 | 6.7% |  | 0.8 | 0.6% |
| Vilke GM et al | 2000 | 39.7% |  |  |  | 0.8 | 1.1 | 5.2% |  |  |  |
| Turedi S et al | 2008 | 0.0% |  | 1.0 | 19.6% |  |  |  |  |  |  |
| Mikhail GM et al | 1992 | 1.8% |  |  |  | 13.0 | 0.9 | 7.1% | 0.0 | 1.0 | 2.7% |
| Jeret JS et al | 1992 | 3.7% |  |  |  | 1.8 | 1.0 | 9.4% | 0.0 | 1.0 | 0.3% |
| Falimirski ME et al | 2003 | 2.1% | 0.0 | 1.2 | 12.1% |  |  |  |  |  |  |
| Ibanez J et al | 2004 | 1.0% |  |  |  | 7.0 | 1.0 | 7.5% |  |  |  |
| Mower WR et al | 2005 | 28.1% |  |  |  | 2.5 | 0.5 | 6.7% |  |  |  |
| Duus BR et al | 1993 | 2.6% |  |  |  | 4.4 | 0.9 | 0.5% |  |  |  |
| Nelson JB et al | 1992 | 39.7% | 1.9 | 0.5 | 15.3% |  |  |  |  |  |  |
| Schynoll W et al | 1993 | 47.3% |  |  |  | 1.6 | 0.5 | 12.1% |  |  |  |
| Lee ST et al | 1995 | 0.8% |  |  |  |  | 0.5 | 1.6% |  |  |  |
| Fabbri A et al | 2003 | 8.1% |  |  |  |  |  |  |  |  |  |
| Barrow A et al | 2012 | 3.6% | 5.5 | 0.8 | 2.2% |  |  |  |  |  |  |
| Thiruppathy et al | 2004 | 3.7% | 2.8 | 1.0 | 38.8% |  |  |  |  |  |  |

| **r) Older age** |  | | | | | | | | | | | |
| --- | --- | --- | --- | --- | --- | --- | --- | --- | --- | --- | --- | --- |
| **Study** | **Age** | **Year** | **Prev risk factor** | **PLR CT** | **NLR CT** | **Prev CT** | **PLR ICI** | **NLR ICI** | **Prev ICI** | **PLR NS** | **NLR NS** | **Prev NS** |
| Haydel MJ et al | >60 | 2000 | 8.1% |  |  |  | 2.2 | 0.9 | 6.9% |  |  |  |
| Fabbri A et al | >75 | 2010 | 26.0% |  |  |  | 1.6 | 0.8 | 6.2% |  |  |  |
| Dunham C et al | >60 | 1996 | 9.8% |  |  |  | 2.4 | 0.9 | 7.2% |  |  |  |
| Borczuk P et al | >60 | 1995 | 19.5% | 1.8 | 0.8 | 8.2% |  |  |  |  |  |  |
| Saboori M et al | >60 | 2007 | 5.9% |  |  |  | 2.0 | 0.9 | 6.7% | 0.0 | 1.1 | 0.6% |
| Stiell IG et al | >65 | 2001 | 11.4% |  |  |  | 3.0 | 0.8 | 8.1% |  |  |  |
| Turedi S et al | >60 | 2008 | 25.0% | 0.4 | 1.2 | 19.6% |  |  |  |  |  |  |
| Munoz-Sanchez MA et al | >54 | 2005 | 10.9% |  |  |  | 2.4 | 0.9 | 37.2% |  |  |  |
| Ibanez J et al | >65 | 2004 | 29.4% |  |  |  | 1.6 | 0.8 | 7.5% |  |  |  |
| Mower WR et al | >65 | 2005 | 14.1% |  |  |  | 1.4 | 0.9 | 6.7% |  |  |  |
| Lee ST et al | >60 | 1995 | 12.3% |  |  |  | 2.7 | 0.8 | 1.5% |  |  |  |
| Arienta C et al | >60 | 1997 | 21.9% |  |  |  | 1.9 | 0.8 | 10.6% |  |  |  |
| Stiell IG et al | >65 | 2005 | 10.6% |  |  |  | 3.0 | 0.8 | 8.3% |  |  |  |
| Kotlyar S et al | >60 | 2010 | 30.6% | 1.0 | 1.0 | 6.4% |  |  |  |  |  |  |
| Kisat et al | >65 | 2011 | 16.3% | 2.3 | 0.8 | 29.2% |  |  |  | 1.7 | 0.9 | 4.2% |
| Zongo et al | >65 | 2012 | 44.2% | 1.0 | 1.0 | 7.1% |  |  |  |  |  |  |
| Barrow A et al | >65 | 2012 | 4.2% | 4.7 | 0.9 | 2.2% |  |  |  |  |  |  |
| Ono K et al | >65 | 2007 | 33.1% | 1.5 | 0.8 | 4.7% |  |  |  |  |  |  |
| Moore MM et al | >65 | 2012 | 24.9% |  |  |  |  |  |  | 2.5 | 0.5 | 1.3% |
| Fabbri A et al | >65 | 2008 | 37.5% |  |  |  | 1.4 | 0.8 | 64.7% |  |  |  |

| **s) GCS < 15** |  | | | | | | | | | | |
| --- | --- | --- | --- | --- | --- | --- | --- | --- | --- | --- | --- |
| **Study** | **Year** | **Prev risk factor** | **PLR CT** | **NLR CT** | **Prev CT** | **PLR ICI** | **NLR ICI** | **Prev ICI** | **PLR NS** | **NLR NS** | **Prev NS** |
| gomez PA et al | 1996 | 5.3% |  |  |  | 1.4 | 0.7 | 50.8% | 11.2 | 0.5 | 1.2% |
| Abdul Latip LS et al | 2003 | 53.2% |  |  |  | 2.5 | 0.4 | 53.2% |  |  |  |
| Fabbri A et al | 2010 | 5.7% |  |  |  | 33.9 | 0.4 | 6.2% |  |  |  |
| Jacobs B et al | 2010 | 17.0% | 1.9 | 0.8 | 19.5% | 2.5 | 0.7 | 14.0% |  |  |  |
| Feuerman T et al | 1988 | 36.7% |  |  |  | 1.9 | 0.6 | 14.2% | 2.1 | 0.4 | 2.1% |
| Dacey RG et al | 1986 | 12.8% |  |  |  | 6.6 | 0.3 | 3.8% | 4.8 | 0.5 | 3.0% |
| Dunham C et al | 1996 | 27.1% |  |  |  | 2.6 | 0.5 | 6.3% |  |  |  |
| Teasdale GM et al | 1990 | 13.7% |  |  |  |  |  |  | 14.9 | 0.2 | 10.3% |
| Borczuk P et al | 1995 | 16.4% | 2.8 | 0.7 | 8.2% | 2.8 | 0.7 | 7.8% | 5.8 | 0.1 | 0.8% |
| Mack LR et al | 2003 | 15.0% |  |  |  | 1.1 | 1.0 | 14.3% |  |  |  |
| Shackford SR et al | 1992 | 31.3% |  |  |  | 1.3 | 0.9 | 16.9% | 1.5 | 0.8 | 4.2% |
| Stiell IG et al | 2001 | 20.2% |  |  |  | 3.1 | 0.6 | 8.1% |  |  |  |
| Turedi S et al | 2008 | 10.8% | 11.1 | 0.6 | 19.6% |  |  |  |  |  |  |
| Mikhail GM et al | 1992 | 1.8% |  |  |  | 0.0 | 1.0 | 7.1% | 0.0 | 1.0 | 2.7% |
| Harad FT et al | 1992 | 16.9% |  |  |  | 1.4 | 0.9 | 18.2% |  |  |  |
| Culotta V et al | 1995 | 28.8% |  |  |  | 2.4 | 0.5 | 8.4% | 2.4 | 0.5 | 0.9% |
| Munoz-Sanchez MA et al | 2005 | 14.1% |  |  |  | 5.7 | 0.7 | 37.2% |  |  |  |
| Ibanez J et al | 2004 | 4.6% |  |  |  | 6.1 | 0.8 | 7.5% |  |  |  |
| Hsiang JNK et al | 1997 | 13.5% | 2.5 | 0.8 | 21.7% |  |  |  | 3.0 | 0.7 | 3.1% |
| Arienta C et al | 1997 | 0.8% |  |  |  | 64.3 | 0.7 | 0.9% | 82.4 | 0.4 | 0.2% |
| Livingston DH et al | 1991a | 18.0% |  |  |  | 2.7 | 0.7 | 13.5% |  |  |  |
| Schynoll W et al | 1993 | 19.3% |  |  |  | 3.6 | 0.5 | 12.1% |  |  |  |
| Stein SC et al | 1992 | 27.4% | 1.9 | 0.7 | 17.2% |  |  |  |  |  |  |
| Murshid WR et al | 1998 | 3.0% |  |  |  | 54.4 |  | 4.9% | 34.6 | 0.3 | 1.9% |
| Stiell IG et al | 2005 | 32.7% | 1.9 | 0.6 | 12.1% | 1.9 | 0.6 | 8.5% | 2.4 | 0.3 | 1.4% |
| Biberthaler 2006 | 2006 | 12.0% | 3.1 | 0.8 | 7.1% |  |  |  |  |  |  |
| Muller 2007 | 2007 | 20.4% |  |  |  | 2.7 | 0.6 | 9.3% |  |  |  |
| Mussack 2002 | 2002 | 7.2% | 56.8 | 0.5 | 13.7% |  |  |  |  |  |  |
| Kotlyar S et al | 2010 | 10.1% | 3.7 | 0.7 | 6.4% |  |  |  |  |  |  |
| Zongo et al | 2012 | 24.0% | 1.4 | 0.9 | 7.1% |  |  |  |  |  |  |
| Barrow A et al | 2012 | 3.4% | 2.8 | 0.9 | 2.2% |  |  |  |  |  |  |
| Thiruppathy et al | 2004 | 20.5% | 2.8 | 0.8 | 38.8% |  |  |  | 1.7 | 0.8 | 7.1% |
| Ono K et al | 2007 | 14.3% | 2.7 | 0.7 | 4.7% |  |  |  |  |  |  |

| **t) GCS 13** |  | | | | | | | | | | |
| --- | --- | --- | --- | --- | --- | --- | --- | --- | --- | --- | --- |
| **Study** | **Year** | **Prev risk factor** | **PLR CT** | **NLR CT** | **Prev CT** | **PLR ICI** | **NLR ICI** | **Prev ICI** | **PLR NS** | **NLR NS** | **Prev NS** |
| gomez PA et al | 1996 | 1.8% |  |  |  | 1.9 | 0.8 | 50.8% | 29.7 | 0.6 | 1.2% |
| Jacobs B et al | 2010 | 4.7% | 3.5 | 0.9 | 19.5% | 4.4 | 0.9 | 14.0% |  |  |  |
| Feuerman T et al | 1988 | 9.1% |  |  |  | 3.7 | 0.8 | 14.2% | 2.9 | 0.8 | 2.1% |
| Dacey RG et al | 1986 | 3.0% |  |  |  |  |  |  | 16.4 | 0.7 | 3.0% |
| Dunham C et al | 1996 | 5.7% |  |  |  | 5.0 | 0.8 | 6.3% |  |  |  |
| Borczuk P et al | 1995 | 2.8% | 4.2 | 0.9 | 8.2% | 4.5 | 0.9 | 7.8% | 10.6 | 0.7 | 0.8% |
| Mack LR et al | 2003 | 3.0% |  |  |  | 2.0 | 1.0 | 14.3% |  |  |  |
| Shackford SR et al | 1992 | 8.0% |  |  |  | 2.4 | 0.9 | 16.9% | 3.5 | 0.8 | 4.2% |
| Stiell IG et al | 2001 | 3.5% |  |  |  | 7.8 | 0.8 | 8.1% |  |  |  |
| Turedi S et al | 2008 | 5.8% | 24.6 | 0.8 | 19.6% |  |  |  |  |  |  |
| Harad FT et al | 1992 | 3.6% |  |  |  | 1.7 | 1.0 | 18.2% |  |  |  |
| Culotta V et al | 1995 | 5.2% |  |  |  | 4.2 | 0.9 | 8.4% | 5.1 | 0.8 | 0.9% |
| Hsiang JNK et al | 1997 | 3.3% | 4.9 | 0.9 | 21.7% |  |  |  | 7.8 | 0.8 | 3.1% |
| Schynoll W et al | 1993 | 2.7% |  |  |  | 2.9 | 1.0 | 12.1% |  |  |  |
| Stein SC et al | 1992 | 7.8% | 2.9 | 0.9 | 17.2% |  |  |  |  |  |  |
| Stein SC et al | 1990 | 9.4% | 3.2 | 0.8 | 17.6% |  |  |  |  |  |  |
| Murshid WR et al | 1998 | 1.7% |  |  |  | 87.4 | 0.7 | 4.9% | 62.1 | 0.5 | 1.9% |
| Stiell IG et al | 2005 | 4.2% |  |  |  | 4.7 | 0.9 | 8.9% |  |  |  |
| Biberthaler 2006 | 2006 | 2.9% | 2.5 | 1.0 | 7.7% |  |  |  |  |  |  |
| Muller 2007 | 2007 | 7.1% |  |  |  | 4.4 | 0.8 | 9.3% |  |  |  |
| Zongo et al | 2012 | 2.5% | 2.9 | 1.0 | 7.1% |  |  |  |  |  |  |
| Thiruppathy et al | 2004 | 8.4% | 3.0 | 0.9 | 38.8% |  |  |  | 2.4 | 0.9 | 7.1% |

| **u) Deterioration of GCS** |  | | | | | | | | | | |
| --- | --- | --- | --- | --- | --- | --- | --- | --- | --- | --- | --- |
| **Study** | **Year** | **Prev risk factor** | **PLR CT** | **NLR CT** | **Prev CT** | **PLR ICI** | **NLR ICI** | **Prev ICI** | **PLR NS** | **NLR NS** | **Prev NS** |
| Stiell IG et al | 2001 | 2.9% |  |  |  | 16.0 | 0.8 | 8.1% |  |  |  |
| Stiell IG et al | 2005 | 10.6% |  |  |  | 3.5 | 0.8 | 8.3% |  |  |  |

| **v) Intoxication** |  | | | | | | | | | | |
| --- | --- | --- | --- | --- | --- | --- | --- | --- | --- | --- | --- |
| **Study** | **Year** | **Prev risk factor** | **PLR CT** | **NLR CT** | **Prev CT** | **PLR ICI** | **NLR ICI** | **Prev ICI** | **PLR NS** | **NLR NS** | **Prev NS** |
| Haydel MJ et al | 2000 | 34.6% |  |  |  | 1.9 | 0.6 | 6.9% |  |  |  |
| Smits M et al | 2007 | 42.7% |  |  |  | 0.7 | 1.2 | 7.6% |  |  |  |
| Fabbri A et al | 2010 | 6.1% |  |  |  | 3.1 | 0.9 | 6.2% |  |  |  |
| Nagy KK et al | 1999 | 21.1% | 0.7 | 1.1 | 3.3% |  |  |  |  |  |  |
| Dunham C et al | 1996 | 38.2% |  |  |  | 0.9 | 1.0 | 6.3% |  |  |  |
| Borczuk P et al | 1995 | 30.9% | 0.8 | 1.1 | 8.2% |  |  |  |  |  |  |
| Mack LR et al | 2003 | 6.8% |  |  |  | 3.0 | 0.9 | 14.3% |  |  |  |
| Stiell IG et al | 2001 | 12.3% |  |  |  | 1.5 | 0.9 | 8.1% |  |  |  |
| Ibanez J et al | 2004 | 6.0% |  |  |  | 1.0 | 1.0 | 7.5% |  |  |  |
| Mower WR et al | 2005 | 24.4% |  |  |  | 0.8 | 1.1 | 6.7% |  |  |  |
| Schynoll W et al | 1993 | 12.9% |  |  |  | 1.6 | 0.9 | 12.1% |  |  |  |
| Stiell IG et al | 2005 | 13.7% |  |  |  | 1.3 | 1.0 | 8.0% |  |  |  |
| Kotlyar S et al | 2010 | 24.3% | 1.3 | 0.9 | 6.4% |  |  |  |  |  |  |
| Zongo et al | 2012 | 24.0% | 1.1 | 1.0 | 7.1% |  |  |  |  |  |  |
| Barrow A et al | 2012 | 26.2% | 2.1 | 0.6 | 2.2% |  |  |  |  |  |  |
| Ono K et al | 2007 | 27.7% | 1.3 | 0.9 | 4.7% |  |  |  |  |  |  |

| **w) Physical evidence of trauma above the clavicles** |  | | | | | | | | | | |
| --- | --- | --- | --- | --- | --- | --- | --- | --- | --- | --- | --- |
| **Study** | **Year** | **Prev risk factor** | **PLR CT** | **NLR CT** | **Prev CT** | **PLR ICI** | **NLR ICI** | **Prev ICI** | **PLR NS** | **NLR NS** | **Prev NS** |
| Haydel MJ et al | 2000 | 65.0% |  |  |  | 1.4 | 0.3 | 6.9% |  |  |  |
| Fabbri A et al | 2010 | 69.0% |  |  |  | 1.1 | 0.7 | 6.2% |  |  |  |
| Dunham C et al | 1996 | 35.3% |  |  |  | 2.0 | 0.5 | 6.3% |  |  |  |
| Holmes JF et al | 1997 | 70.1% |  |  |  | 1.4 | 0.2 | 13.3% | 1.4 | 0.0 | 1.5% |
| Brewer ES et al | 2010 | 76.6% |  |  |  | 0.9 | 1.6 | 29.1% |  |  |  |
| Stiell IG et al | 2005 | 35.0% |  |  |  | 1.7 | 0.7 | 3.7% |  |  |  |
| Kotlyar S et al | 2010 | 38.7% | 1.6 | 0.7 | 6.4% |  |  |  |  |  |  |
| Barrow A et al | 2012 | 45.1% | 0.2 | 1.7 | 2.2% |  |  |  |  |  |  |

| **x) Coagulopathy or anticoagulation** |  | | | | | | | | | | |
| --- | --- | --- | --- | --- | --- | --- | --- | --- | --- | --- | --- |
| **Study** | **Year** | **Prev risk factor** | **PLR CT** | **NLR CT** | **Prev CT** | **PLR ICI** | **NLR ICI** | **Prev ICI** | **PLR NS** | **NLR NS** | **Prev NS** |
| Haydel MJ et al | 2000 | 0.2% |  |  |  | na | 1.0 | 6.9% |  |  |  |
| Fabbri A et al | 2010 | 3.0% |  |  |  | 6.2 | 0.9 | 6.2% |  |  |  |
| Smits M et al | 2007 | 2.6% |  |  |  | 2.2 | 1.0 | 6.3% |  |  |  |
| Borczuk P et al | 1995 | 3.5% | 2.1 | 1.0 | 8.2% |  |  |  |  |  |  |
| Mack LR et al | 2003 | 13.5% |  |  |  | 1.2 | 1.0 | 14.3% |  |  |  |
| Saboori M et al | 2007 | 0.1% |  |  |  | 0.0 | 1.0 | 6.7% | 0.0 | 1.0 | 0.6% |
| Turedi S et al | 2008 | 0.0% |  | 1.0 | 19.6% |  |  |  |  |  |  |
| Ibanez J et al | 2004 | 11.4% |  |  |  | 3.3 | 0.7 | 7.5% |  |  |  |
| Mower WR et al | 2005 | 3.6% |  |  |  | 1.4 | 1.0 | 6.7% |  |  |  |
| Kotlyar S et al | 2010 | 6.1% | 0.0 | 1.1 | 6.4% |  |  |  |  |  |  |
| Zongo et al | 2012 | 27.9% | 2.9 | 0.4 | 7.1% |  |  |  |  |  |  |
| Barrow A et al | 2012 | 5.2% | 8.0 | 0.7 | 2.2% |  |  |  |  |  |  |
| Moore MM et al | 2012 | 8.8% |  |  |  |  |  |  | 4.2 | 0.7 | 1.3% |
| Claudia C et al | 2010 | 5.3% |  |  |  | 2.8 | 0.9 | 6.3% |  |  |  |

| **y) Anti-platelet medication** |  | | | | | | | | | | |
| --- | --- | --- | --- | --- | --- | --- | --- | --- | --- | --- | --- |
| **Study** | **Year** | **Prev risk factor** | **PLR CT** | **NLR CT** | **Prev CT** | **PLR ICI** | **NLR ICI** | **Prev ICI** | **PLR NS** | **NLR NS** | **Prev NS** |
| Fabbri A et al | 2010 | 9.6% |  |  |  | 2.3 | 0.9 | 6.2% |  |  |  |
| Mack LR et al | 2003 | 21.8% |  |  |  | 0.4 | 1.2 | 14.3% |  |  |  |
| Kotlyar S et al | 2010 | 10.4% | 1.3 | 1.0 | 6.4% |  |  |  |  |  |  |

| **z) Any fall** |  | | | | | | | | | | |
| --- | --- | --- | --- | --- | --- | --- | --- | --- | --- | --- | --- |
| **Study** | **Year** | **Prev risk factor** | **PLR CT** | **NLR CT** | **Prev CT** | **PLR ICI** | **NLR ICI** | **Prev ICI** | **PLR NS** | **NLR NS** | **Prev NS** |
| Smits M et al | 2007 | 21.0% |  |  |  | 1.8 | 0.8 | 14.6% |  |  |  |
| Dunham C et al | 1996 | 11.4% |  |  |  | 1.3 | 1.0 | 6.3% |  |  |  |
| Borczuk P et al | 1995 | 30.5% | 1.2 | 0.9 | 8.2% |  |  |  |  |  |  |
| Stiell IG et al | 2001 | 30.9% |  |  |  | 1.4 | 0.8 | 8.1% |  |  |  |
| Harad FT et al | 1992 | 18.2% |  |  |  | 0.9 | 1.0 | 18.2% | 1.0 | 1.0 | 3.6% |
| Munoz-Sanchez MA et al | 2005 | 53.8% |  |  |  | 0.7 | 1.5 | 37.2% |  |  |  |
| Ibanez J et al | 2004 | 51.6% |  |  |  |  |  |  |  |  |  |
| Miller EC et al | 1996 | 26.8% | 0.9 | 1.0 | 6.1% |  |  |  |  |  |  |
| Livingston DH et al | 1991a | 5.4% |  |  |  | 1.3 | 1.0 | 13.5% |  |  |  |
| Schynoll W et al | 1993 | 55.3% |  |  |  | 1.0 | 1.0 | 12.1% |  |  |  |
| Brewer ES et al | 2010 | 91.5% |  |  |  | 0.9 | 2.4 | 29.1% |  |  |  |
| Gutman MB et al | 1992 | 34.5% |  |  |  | 2.3 | 0.6 | 26.9% |  |  |  |
| Murshid WR et al | 1998 | 53.6% |  |  |  |  |  |  | 1.4 | 0.5 | 1.9% |
| Kotlyar S et al | 2010 | 44.8% | 1.5 | 0.6 | 6.4% |  |  |  |  |  |  |
| Kisat et al | 2011 | 21.2% | 2.2 | 0.8 | 29.2% |  |  |  | 1.7 | 0.8 | 4.2% |
| Zongo et al | 2012 | 38.1% | 1.0 | 1.0 | 7.1% |  |  |  |  |  |  |
| Ono K et al | 2007 | 36.5% | 0.9 | 1.1 | 4.7% |  |  |  |  |  |  |

| **aa) Fall >1 metre or >5 stairs** |  | | | | | | | | | | |
| --- | --- | --- | --- | --- | --- | --- | --- | --- | --- | --- | --- |
| **Study** | **Year** | **Prev risk factor** | **PLR CT** | **NLR CT** | **Prev CT** | **PLR ICI** | **NLR ICI** | **Prev ICI** | **PLR NS** | **NLR NS** | **Prev NS** |
| Stiell IG et al | 2001 | 10.2% |  |  |  | 1.7 | 0.9 | 8.1% |  |  |  |
| Ono K et al | 2007 | 13.0% | 2.3 | 0.8 | 4.7% |  |  |  |  |  |  |

| **ab) Fall >3 metres or >15 stairs** |  | | | | | | | | | | |
| --- | --- | --- | --- | --- | --- | --- | --- | --- | --- | --- | --- |
| **Study** | **Year** | **Prev risk factor** | **PLR CT** | **NLR CT** | **Prev CT** | **PLR ICI** | **NLR ICI** | **Prev ICI** | **PLR NS** | **NLR NS** | **Prev NS** |
| Stiell IG et al | 2001 | 4.4% |  |  |  | 2.3 | 0.9 | 8.1% |  |  |  |

| **ac) Ejected from vehicle** |  | | | | | | | | | | |
| --- | --- | --- | --- | --- | --- | --- | --- | --- | --- | --- | --- |
| **Study** | **Year** | **Prev risk factor** | **PLR CT** | **NLR CT** | **Prev CT** | **PLR ICI** | **NLR ICI** | **Prev ICI** | **PLR NS** | **NLR NS** | **Prev NS** |
| Smits M et al | 2007 | 1.0% |  |  |  | 2.6 | 1.0 | 7.6% |  |  |  |
| Stiell IG et al | 2001 | 9.5% |  |  |  | 3.2 | 0.8 | 1.3% |  |  |  |

| **ad) Pedestrian or cyclist verses vehicle** |  | | | | | | | | | | |
| --- | --- | --- | --- | --- | --- | --- | --- | --- | --- | --- | --- |
| **Study** | **Year** | **Prev risk factor** | **PLR CT** | **NLR CT** | **Prev CT** | **PLR ICI** | **NLR ICI** | **Prev ICI** | **PLR NS** | **NLR NS** | **Prev NS** |
| Smits M et al | 2007 | 9.4% |  |  |  | 2.7 | 0.9 | 12.8% |  |  |  |
| Stiell IG et al | 2001 | 6.7% |  |  |  | 5.6 | 0.8 | 8.1% |  |  |  |
| Harad FT et al | 1992 | 13.6% |  |  |  | 1.4 | 0.9 | 18.2% | 0.0 | 1.2 | 3.6% |
| Jeret JS et al | 1992 | 14.0% |  |  |  | 2.1 | 0.8 | 9.4% | 0.0 | 1.2 | 0.3% |
| Schynoll W et al | 1993 | 4.9% |  |  |  | 4.5 | 0.9 | 12.1% |  |  |  |

| **ae) Bicycle accident** |  | | | | | | | | | | |
| --- | --- | --- | --- | --- | --- | --- | --- | --- | --- | --- | --- |
| **Study** | **Year** | **Prev risk factor** | **PLR CT** | **NLR CT** | **Prev CT** | **PLR ICI** | **NLR ICI** | **Prev ICI** | **PLR NS** | **NLR NS** | **Prev NS** |
| Dunham C et al | 1996 | 1.3% |  |  |  | 1.9 | 1.0 | 6.3% |  |  |  |
| Borczuk P et al | 1995 | 1.9% | 3.2 | 1.0 | 8.2% |  |  |  |  |  |  |
| Stiell IG et al | 2001 | 6.6% |  |  |  | 0.6 | 1.0 | 8.1% |  |  |  |
| Gutman MB et al | 1992 | 3.1% |  |  |  | 0.6 | 1.0 | 26.9% |  |  |  |
| Kisat et al | 2011 | 18.8% | 1.3 | 0.9 | 29.2% |  |  |  |  |  |  |

| **af) Motorcycle accident** |  | | | | | | | | | | |
| --- | --- | --- | --- | --- | --- | --- | --- | --- | --- | --- | --- |
| **Study** | **Year** | **Prev risk factor** | **PLR CT** | **NLR CT** | **Prev CT** | **PLR ICI** | **NLR ICI** | **Prev ICI** | **PLR NS** | **NLR NS** | **Prev NS** |
| Dunham C et al | 1996 | 7.3% |  |  |  | 1.0 | 1.0 | 6.3% |  |  |  |
| Borczuk P et al | 1995 | 2.5% | 3.2 | 1.0 | 8.2% |  |  |  |  |  |  |
| Stiell IG et al | 2001 | 1.7% |  |  |  | 0.4 | 1.0 | 8.1% |  |  |  |
| Gutman MB et al | 1992 | 2.5% |  |  |  | 0.4 | 1.0 | 26.9% |  |  |  |

| **ag) Motor vehicle accident** |  | | | | | | | | | | |
| --- | --- | --- | --- | --- | --- | --- | --- | --- | --- | --- | --- |
| **Study** | **Year** | **Prev risk factor** | **PLR CT** | **NLR CT** | **Prev CT** | **PLR ICI** | **NLR ICI** | **Prev ICI** | **PLR NS** | **NLR NS** | **Prev NS** |
| Abdul Latip LS et al | 2003 | 75.5% |  |  |  |  |  |  | 0.6 | 3.0 | 20.2% |
| Dunham C et al | 1996 | 58.9% |  |  |  | 0.7 | 1.4 | 6.3% |  |  |  |
| Borczuk P et al | 1995 | 33.4% | 0.6 | 1.2 | 8.2% |  |  |  |  |  |  |
| Mack LR et al | 2003 | 4.5% |  |  |  | 1.2 | 1.0 | 14.3% |  |  |  |
| Stiell IG et al | 2001 | 25.8% |  |  |  | 0.6 | 1.1 | 8.1% |  |  |  |
| Harad FT et al | 1992 | 42.7% |  |  |  | 0.6 | 1.4 | 18.2% | 0.0 | 1.8 | 3.6% |
| Jeret JS et al | 1992 | 37.9% |  |  |  | 0.6 | 1.3 | 9.4% | 0.0 | 1.6 | 0.3% |
| Ibanez J et al | 2004 | 31.1% |  |  |  |  |  |  |  |  |  |
| Miller EC et al | 1996 | 40.9% | 1.0 | 1.0 | 6.1% |  |  |  |  |  |  |
| Livingston DH et al | 1991a | 33.3% |  |  |  | 0.8 | 1.1 | 13.5% |  |  |  |
| Schynoll W et al | 1993 | 21.6% |  |  |  | 1.2 | 1.0 | 12.1% |  |  |  |
| Brewer ES et al | 2010 | 8.5% |  |  |  | 2.4 | 0.9 | 29.1% |  |  |  |
| Gutman MB et al | 1992 | 26.5% |  |  |  | 0.3 | 1.3 | 26.9% |  |  |  |
| Murshid WR et al | 1998 | 37.0% |  |  |  |  |  |  | 0.4 | 1.3 | 1.9% |
| Kotlyar S et al | 2010 | 30.3% | 0.3 | 1.3 | 6.4% |  |  |  |  |  |  |
| Kisat et al | 2011 | 59.5% | 0.6 | 1.7 | 29.2% |  |  |  | 0.7 | 1.5 | 4.2% |
| Zongo et al | 2012 | 14.2% | 1.0 | 1.0 | 7.1% |  |  |  |  |  |  |
| Ono K et al | 2007 | 5.3% | 0.8 | 1.0 | 4.7% |  |  |  |  |  |  |

| **ah) Elevated S100B levels** |  | | | | | | | | | | |
| --- | --- | --- | --- | --- | --- | --- | --- | --- | --- | --- | --- |
| **Study** | **Year** | **Prev risk factor** | **PLR CT** | **NLR CT** | **Prev CT** | **PLR ICI** | **NLR ICI** | **Prev ICI** | **PLR NS** | **NLR NS** | **Prev NS** |
| Ingebrigtsen T et al | 2000 | 37.9% | 3.0 | 0.1 | 9.9% | 2.6 | 0.2 | 5.5% | 2.7 | 0.0 | 0.5% |
| Bazarian J et al | 2006 | 88.4% | 0.8 | 2.3 | 4.7% | 1.1 | 0.0 | 3.5% |  |  |  |
| Nygren de boussard C et al | 2004 | 56.1% | 1.9 | 0.0 | 6.1% |  |  |  |  |  |  |
| Poli-de-Figueiredo LF et al | 2006 | 82.0% | 1.3 | 0.0 | 12.0% |  |  |  |  |  |  |
| Muller K et al | 2007 | 71.2% |  |  |  | 1.4 | 0.2 | 9.3% |  |  | 0.0% |
| Biberthaler P et al | 2006 | 72.3% | 1.4 | 0.0 | 7.1% |  |  |  | 1.4 | 0.0 | 0.8% |
| Biberthaler P et al | 2002 | 64.4% | 1.9 | 0.0 | 23.1% |  |  |  |  |  |  |
| Biberthaler P et al | 2001 | 71.2% | 1.7 | 0.0 | 28.8% |  |  |  | 1.4 | 0.0 | 1.9% |
| Mussack T et al | 2002 | 56.8% | 2.0 | 0.0 | 13.7% | 1.9 | 0.0 | 7.9% | 1.8 | 0.0 | 2.9% |
| Bouvier D et al | 2009 | 72.4% | 1.5 | 0.0 | 15.2% |  |  |  | 1.4 | 0.0 | 1.0% |
| Morochovic R et al | 2009 | 72.5% |  |  |  | 1.2 | 0.6 | 17.6% |  |  |  |
| Kotlyar S et al | 2010 | 88.0% | 1.1 | 0.3 | 13.9% |  |  |  |  |  |  |
| Muller B et al | 2010 | 70.0% | 1.3 | 0.4 | 9.4% |  |  |  |  |  |  |
| Zongo et al | 2012 | 81.3% | 1.2 | 0.0 | 7.1% |  |  |  |  |  |  |

| **ai) S100B levels above 0.10 µg/L** |  | | | | | | | | | | |
| --- | --- | --- | --- | --- | --- | --- | --- | --- | --- | --- | --- |
| **Study** | **Year** | **Prev risk factor** | **PLR CT** | **NLR CT** | **Prev CT** | **PLR ICI** | **NLR ICI** | **Prev ICI** | **PLR NS** | **NLR NS** | **Prev NS** |
| Bazarian J et al | 2006 | 88.4% | 0.8 | 2.3 | 4.7% |  |  |  |  |  |  |
| Nygren de boussard C et al | 2004 | 56.1% | 1.9 | 0.0 | 6.1% |  |  |  |  |  |  |
| Poli-de-Figueiredo LF et al | 2006 | 82.0% | 1.3 | 0.0 | 12.0% |  |  |  |  |  |  |
| Muller K et al | 2007 | 71.2% |  |  |  | 1.4 | 0.2 | 9.3% |  |  |  |
| Biberthaler P et al | 2006 | 72.3% | 1.4 | 0.0 | 7.1% |  |  |  |  |  |  |
| Biberthaler P et al | 2001 | 71.2% | 1.7 | 0.0 | 28.8% |  |  |  |  |  |  |
| Bouvier D et al | 2009 | 72.4% | 1.5 | 0.0 | 15.2% |  |  |  |  |  |  |
| Morochovic R et al | 2009 | 72.5% |  |  |  | 1.2 | 0.6 | 17.6% |  |  |  |
