# Supplementary material for: Scandinavian guidelines for initial management of minimal, mild and moderate head injuries in adults: an evidence and consensus-based update
Source: BMC Med. 2013 Feb 25;11:50. doi: 10.1186/1741-7015-11-50 (PMC3621842; doi:10.1186/1741-7015-11-50)
Supplement: Additional file 2 — Table S2. Evidentiary table of studies with reference to the clinical question: 'Which adult patients with minimal, mild and moderate head injury need a head CT and which patients may be directly discharged?'. CT = computed tomography. [file 1741-7015-11-50-S2.DOC]

Additional file 2: Table S2. Evidentiary table of studies with reference to the clinical question: “Which adult patients with minimal, mild and moderate head injury need a head CT and which patients may be directly discharged?”. MHI=Minor Head Injury, P=Prospective, R=Retrospective, GCS=Glasgow Coma Scale, HI=Head Injury, CT=Computed Tomography, LOC=Loss of consciousness, PTA=post-traumatic amnesia, GOS=Glasgow Outcome Score, SF=Scull fracture, SB=Selection bias, VB=Verification bias.

| **Study** | **Year** | **Design** | **n** | **Age** | **GCS** | **Other inclusion criteria** | **Exclusion criteria** | **CT%** | **Follow up** | **Evidence level (CEBM)** | **Limitations and comments** |
| --- | --- | --- | --- | --- | --- | --- | --- | --- | --- | --- | --- |
| Zongo et al | 2012 | P | 1560 | >14 | 13-15 | GCS 13-15, at least one risk factor (LOC, amnesia, rep vomit, sev HA, diz, vertigo, intox, anticoag, age>65) | Severe injury AIS > 2 | 100 |  | 2 |  |
| Barrow A | 2012 | P | 497 | >16 | 14-15 | >4 hours after injury |  | 30 | Poor attempt, only 14% contactable | 4 | VB, SB |
| Moore MM et al | 2012 | R | 7678 | >14 | 14-15 | Blunt HI |  | na | Records | 4 | SB |
| Kisat M et al | 2011 | R | 83566 | >15 | 15 | Blunt HI. | Penetrating injuries, burns, missing data, age < 16, GCS <15, No CT reports | 100 |  | 3 | SB |
| Fabbri A et al | 2010 | P | 14228 | >9 | 14-15 | Acute HI, <24 hours of event | Unclear history of injury, unstable vital signs, GCS <14, penetrating injuries, discharge against medical advice, duplicate records | 64 | 6 months GOS with patients with lesions + death certificates and databases of all patients | 3 |  |
| Claudia C et al | 2010 | R | 1410 | >17 | 14-15 | GCS 14-15, adults | No other risk factors. No CT done. | 100 |  | 3 | SB |
| Jacobs B et al | 2010 | P | 2784 | >15 | 13-15 | GCS 15 and LOC/PTA/unclear history, persistent or progressive headache, nausea, vomiting, intoxication, seizure, any anticoag incl antiplatlet, confusion, amnesia, focal deficits, age over 60 or under 2, high energy accident, visible trauma above clavicles | Penetrating head injury | 72 | 6 months GOSE at follow-up or via telephone | 3 |  |
| Abdul Rahman YS et al | 2010 | P | 152 | 0-76 | 13-15 | Blunt HI. LOC, vomiting, headache, convulsions and/or amnesia |  | 100 | No | 2 | Probable SB |
| Brewer ES et al | 2010 | R | 141 | 36-101 | 15 | HI with clopidogrel or warfarin therapy and CT |  | 100 | Medical records | 4 | SB |
| Kotlyar S et al | 2010 | P | 346 | >17 | 13-15 | Blunt HI, intent of CT | Major trauma, head trauma > 6 hours to presentation, altered mental status, known prior intracranial pathology, focal neurology | 100 | No | 4 | SB. Different cuf-offs tested |
| Muller B et al | 2010 | P | 233 | > 15 | 13-15 | Blunt HI | Multiple injuries, cancer, stroke, neurological disease, intracranial bleeds > 5mm, multiple bleeds, coaulopathy, platelet inhibitors, intoxication | 100 | No | 2 | Excluded some ICI. S100B cut-off of 0,105µg/L |
| Bouvier D et al | 2009 | P | 105 | > 17 | 13-15 | Risk factors | <18 years, pregnancy, multiple injuries | 100 | No | 2 | S100B cut-off 0.10µg/L |
| Morochovic R et al | 2009 | P | 102 | 12-84 | 13-15 | LOC, amnesia or risk factors | Unknown history, acute non-trauma intracererbal injuries | 100 | Short-term hospital follow-up | 2 | SB, S100B cut-off 0.10µg/L |
| Turedi S et al | 2008 | P | 240 | All | 13-15 | Blunt HI, LOC < 15 min or post-traumatic amnesia <1 hr |  | 100 | No | 2 | SB, 2 cohorts |
| Fabbri A et al | 2008 | P | 309 | >9 | 9 to 13 | <24 hours of HI, >9years | Hypotension from extracran injuries, CPR, penetrating HI, intubation | 100 | 6 months GOS with patients with lesions + death certificates and databases of all patients | 2 |  |
| Smits M et al | 2007 | P | 3181 | >15 | 13-15 | <24 hrs after head injury, GCS 15 + risk factor (LOC, amnesia, posttraumatic seizure, vomiting, severe headache, intoxication, anticoagulant (not aspirin)/coagulopathy, external evidence of injury above clavicles and neurological deficit) | Transfer from other hospitals, contraindication for CT, concurrent injuries precluding head CT | 100 | Short-term hospital follow-up | 2 |  |
| Saboori M et al | 2007 | P | 682 | >6 | 15 | Blunt HI | >24 hrs after trauma, < 6 years, unclear history, penetrating/open head injury | 100 | 1 week, telephone interview | 2 | SB |
| Muller K et al | 2007 | P | 226 | >18 | 13-15 | LOC/amnesia, GCS, sampling <12hours, CT <12hours | Focal deficits, multiple injuries, renal/liver disease, age <18 years | 100 | No | 2 | SB, S100B cut-off 0.10µg/L |
| Bazarian J et al | 2006 | P | 96 | All | 14-15 | LOC<30 min/amnesia<24houts/altered mental state, sampling <4hours | Confounding conditions related to S100 (Downs, schizophrenia) | 100 | 3 month follow-up | 4 | SB, S100B cut-off 0.10µg/L |
| Poli-de-Figueiredo LF et al | 2006 | P | 50 | na | 13-15 | Amnesia/LOC/nausea/vomiting/vertigo/severe headache | Focal deficits | 100 | No | 2 | S100B cut-off 0.10µg/L |
| Biberthaler P et al | 2006 | P | 1309 | >18 | 13-15 | HI within 3 hours, amnesia/LOC/nausea/vomiting/vertigo/severe headache/dizziness/intox/antikoag/age>60 | <18 years, pregnancy, multiple injuries | 100 | No | 2 | S100B cut-off 0.10µg/L |
| Munoz-Sanchez MA et al | 2005 | P | 156 | All | 14-15 | SF | GCS 15 with no neurological sings or symptoms | 100 | No | 3 | SB |
| Mower WR et al | 2005 | P | 13728 | na | All | Blunt head injury where CT was ordered | Non-blunt HI, delayed presentation | 100 | No | 4 | SB |
| Stiell IG et al | 2005 | P | 2707 | >15 | 13-15 | Blunt HI with LOC/amnesia/disorientation, HI within 24 hours | <16 years, minimal HI (no LOC/amnesia/disorientation), no clear history of trauma, penetrating injury, depressed SF, focal neurology, seizure before HI, bleeding disorder/anticoagulants, reassessment, pregnancy | 80 | Patients with no CT had telephone interview after 14 days, CT if symtoms | 1 | SB |
| Ibanez J et al | 2004 | P | 1101 | >14 | 14-15 |  | Referral from other centers | 100 | No | 2 |  |
| Nygren de boussard C et al | 2004 | P | 66 | 15-65 | 14-15 | Blunt HI <24hours, LOC/amnesia | Amnesia >24hours, LOC >30min, no clear history, other major injury, neurological disorder | 100 | 1, 7 and 14 days | 2 | Possible SB, S100B cut-off 0.10µg/L. MRI also done |
| Abdul Latip LS et al | 2003 | P | 94 | >11 | 13-15 | Blunt HI | Medical illness, CVI, anti-coagulants, brain surgery, facial fracture | 100 | Short-term hospital follow-up | 2 | Probable SB |
| Mack LR et al | 2003 | R | 133 | >64 | 13-15 | Head trauma | GCS < 13, not head trauma | 100 | Medical records | 4 | SB |
| Falimirski ME et al | 2003 | P | 331 | >15 | 14-15 | Blunt HI, LOC/PTA | GCS <14, transfer from outside institution with CT scan | 100 | Yes | 2 | SB |
| Fabbri A et al | 2003 | P | d | >9 | 14-15 | Within 24 hours of HI | Re-attendance | 47 | 6 months GOS with patients with lesions + death certificates and databases of all patients | 3 | Possible VB. |
| Biberthaler P et al | 2002 | P | 104 | na | 13-15 | Amnesia/LOC/nausea/vomiting/vertigo/severe headache | Focal deficits | 100 | No | 2 | S100B cut-off 0.12µg/L |
| Mussack T et al | 2002 | P | 139 | na | 13-15 | Amnesia/LOC/nausea/vomiting/vertigo | Refusal to CT | 100 | No | 2 | S100B cut-off 0.21µg/L |
| Stiell IG et al | 2001 | P | 3121 | >15 | 13-15 | LOC, amnesia and/or disorientation, injury within 24 hrs | Minimal HI (no LOC/amnesia), age <16, penetrating injury, focal signs, unstable vital signs, seizure before ED, bleeding disorder/coumadin, pregnancy | 67 | 14 days telephone follow up to detect neurological intervention, n=1043 (33%) | 3 |  |
| Biberthaler P et al | 2001 | P | 52 | na | 13-15 | Amnesia/LOC/nausea/vomiting/vertigo/severe headache | Focal deficits | 100 | No | 2 | Probable SB. S100B cut-off 0.10µg/L |
| Haydel MJ et al | 2000 | P | 520 | >2 | 15 | LOC/amnesia, no focal signs, <24 hours | Patients denying CT, concurrent injuries, no LOC/amnesia | 100 | No | 1 | SB |
| Livingston DH et al | 2000 | P | 2152 | > 15 | 14-15 | Blunt HI, LOC/amnesia | GCS<14 | 100 | 4-8, 20 hours and at discharge | 2 | Possible SB |
| Vilke GM et al | 2000 | P | 58 | >13 | 15 | Non-penetrating HI, LOC/amnesia | No LOC or amnesia. Pregnancy, intoxication, major trauma. GCS <15 | 100 | Short-term hospital follow-up | 2 | SB |
| Ingebrigtsen T et al | 2000 | P | 182 | 15-80 | 13-15 | HI with LOC < 10 min, within 12 hours of HI, CT within 24 hours | Focal deficits | 100 | 3 months after injury | 3 | SB. Older detection limit concerning S100B |
| Nagy KK et al | 1999 | P | 1170 | All | 15 | Blunt HI with LOC/amnesia |  | 100 | Short-term hospital follow-up | 2 | SB |
| Murshid WR et al | 1998 | R | 633 | 0-90 | 13-15 | HI within 24 hours |  | 21 | 6 weeks to 7 years | 3 | Unclear follow up, possible VB |
| Holmes JF et al | 1997 | P | 264 | na | 14 | LOC/amnesia, intoxicated patients where HI could not be excluded | >4 hours after trauma | 100 | No | 3 | SB |
| Hsiang JNK et al | 1997 | P | 1360 | >10 | 13-15 |  | Not admitted, age < 11 | 62 | 6 months GOS | 3 | VB |
| Arienta C et al | 1997 | R | 10000 | 6-95 | All | Primary patients | Referral, age < 6 years | 8 | Telephone in most patients (99,7%) | 3 | Probable VB, subgroup analysis |
| Miller EC et al | 1997 | P | 2143 | All | 15 | < 24 hours after HI, LOC/amnesia, normal mental status |  | 100 | Short-term hospital follow-up | 2 | Possible SB |
| Gomez PA et al | 1996 | R | 2484 | >15 | 13-15 | Any blow to the head | GCS < 13 after 4 hr, lucid interval, isolated fasial trauma | 8 | Medical records | 3 | Probable VB |
| Dunham C et al | 1996 | P | 2252 | 14-60 | 13-15 | GCS 15 + amnesia and/or witnessed loss of consciousness | Interhospital transfers | 91 | Short-term hospital follow-up | 2 |  |
| Hung CC et al | 1996 | R | 28500 | All | All | All head injuries |  | na | Medical records | 3 | SB. Possible VB. Data analysis on 16464 patients |
| Miller EC et al | 1996 | P | d | All | 15 | LOC/amnesia, normal mental status |  | 100 | Patients with abnormal CT followed until discaharge | 2 |  |
| Borczuk P et al | 1995 | R | 1448 | >16 | 13-15 | LOC, amnesia, focal deficits, progressive/persistent symptoms, intoxication, dangerous mechanism | Age<17, penetrating injuries | 100 | Medical records | 2 | Possible SB |
| Culotta V et al | 1995 | R | 3370 | na | 13-15 | Non-missile HI, LOC |  | 92 | Medical records | 3 |  |
| Lee ST et al | 1995 | P | 1812 | 16-86 | 15 | Blunt HI. LOC or amnesia <30min | Scalp and soft tissue injury of face | na | 3, 7 days, 1 and 3 months | 4 | SB. ICI+ = neurological deterioration, 23/28 for intracranial hematomas (2 seizures and 3 SIADH) |
| Moran SG et al | 1994 | R | 200 | 6-83 | 13-15 | Patients transported to facility by air ambulance. Scene and emergency room GCS 13-15 |  | 48 | Medical records | 3 | VB, probable SB |
| Murshid WR | 1994 | R | d | 0-80 | 13-15 | Within 24 hours of HI |  | 22 | Medical records | 3 | SB |
| Duus BR et al | 1993 | R | 1876 | All | na | Admission after MHI including cerebral concussion, all able to "walk and talk" at medical contact | Depressed or basilar SF | 1 | Medical records | 4 | SB, probable VB |
| Schynoll W et al | 1993 | P | 264 | All | All | ER patients with blunt HI within 2 weeks with CT | Penetrating HI | 100 | No | 4 |  |
| Shackford SR et al | 1992 | P | 2766 | 0-90 | 13-15 | Isolated head injury, LOC or posttraumatic amnesia | Significant associated non-head injuries | 78 | Short-term hospital follow-up | 3 | Probable SB |
| Mikhail GM et al | 1992 | P | 112 | 15-85 | 13-15 | Blunt HI | Penetrating/open injury | 31 | Non CT patients telephone interview after 4 weeks | 3 |  |
| Harad FT et al | 1992 | P | 497 | na | All | CT scan due to: LOC, amnesia, focal deficits, depressed or open skull fracture, deteriorating mental status or pupillary inequality, GCS<13 |  | 100 | Short-term hospital follow-up | 3 | SB |
| Jeret JS et al | 1992 | P | 712 | >18 | 15 | LOC/amnesia, within 24 hrs of HI | Transfer from other hospitals, <18, penetrating HI | 100 | No | 2 | SB |
| Nelson JB et al | 1992 | P | 131 | >14 | 15 | Trauma patients with head CT | <15 years | 100 | Probable short-term hospital follow-up | 4 | SB |
| Stein SC et al | 1992 | R | 1538 | All | 13-15 | LOC and/or amnesia of any period | Focal deficits | 100 | Medical record review for complications after CT | 2 | Possible SB |
| Ando S et al | 1992 | R | 147 | All | na | HI | Other causes of vomitting (6 patients) | 63 | No | 3 | SB. Data reported only in patients with CT done (n=93) |
| Gutman MB et al | 1992 | P | 1039 | >15 | All | LOC or SF | Age < 16, chronic subdural hematomas | 100 | No | 2 | Possible SB. ICI = operable hematoma |
| Rosenorn J et al | 1991 | P | d | All | na | Admission after MHI including cerebral concussion, all able to "walk and talk" at medical contact | Depressed or basilar SF | na | Medical records | 4 | SB, VB |
| Livingston DH et al | 1991 | P | 111 | >16 | 14-15 |  |  | 100 | Telephone interview next day | 2 | Possible SB |
| Livingston DH et al | 1991 | R | 138 | 2-63 | 14-15 | Blunt HI | Obvious cranial injuries (signs bas or open/depressed SF) | 54 | Medical records | 3 | SB |
| Teasdale GM et al | 1990 | P | 9326 | All | All | Operation of traumatic haematoma vs all patients attending ED during study period | Infants with fluid subdural collections | na | No | 4 | SB |
| Stein SC et al | 1990 | R | 658 | All | 13-15 | LOC or amnesia |  | 100 | Medical records | 2 | Possible SB |
| Feuerman T et al | 1988 | R | 373 | >15 | 13-15 | Within 24 hours |  | 35 | Medical records | 3 |  |
| Servadei F et al | 1988 | P | 98 | > 13 | 14-15 | Recent HI, Skull fracture vs no skull fracture |  | na | 6 months | 4 | SB, probable VB |
| Masters SJ et al | 1987 | P | 7035 | na | All | All HI in 31 ER´s |  | na | 3658 with follow up and 1015 with data from National Death Index | 3 | SB, probable VB |
| Dacey RG et al | 1986 | P | 610 | All | 13-15 | LOC or memory/speech/vision dysfunction | Gunshot wounds | 11 | Hospital course | 2 | VB |
