# Supplementary material for: Scandinavian guidelines for initial management of minimal, mild and moderate head injuries in adults: an evidence and consensus-based update
Source: BMC Med. 2013 Feb 25;11:50. doi: 10.1186/1741-7015-11-50 (PMC3621842; doi:10.1186/1741-7015-11-50)
Supplement: Additional file 3 — Table S3. Modified Quality Assessment of Diagnostic Accuracy Studies (QUADAS) grading of studies referring to the clinical question: 'Which adult patients with minimal, mild and moderate head injury need a head CT and which patients may be directly discharged?'. CT = computed tomography. [file 1741-7015-11-50-S3.DOC]

Additional file 3: Table S3. Modified QUADAS grading of studies referring to the clinical question: “Which adult patients with minimal, mild and moderate head injury need a head CT and which patients may be directly discharged?”. CT=Computed Tomography, ICI=Intracranial injury, NS=Neurosurgery, Y=Yes, N=no, U=Unknown, na=not applicable.

| Study | Year | Data | Low risk of selection bias/spectrum of patients representative | Selection criteria described well | Acceptable reference test, CT | Acceptable reference test, ICI | Acceptable reference test, NS | Did all patients recieve reference test | Did all patients recieve the same reference test | Index test described well | Reference test descibed well | Index test blinded to reference test | Reference test blinded to index test | Same clinical data as in practice | Uninterpretable data reported | Withdrawals explained |
| --- | --- | --- | --- | --- | --- | --- | --- | --- | --- | --- | --- | --- | --- | --- | --- | --- |
| Barrow A | 2012 | data CT | N | Y | U | na | na | N | N | Y | N | N | Y | Y | U | N |
| Moore MM et al | 2012 | data NS | U | Y | na | na | U | Y | N | Y | N | Y | Y | Y | U | N |
| Zongo et al | 2012 | data CT | N | Y | Y | na | N | Y | Y | Y | Y | N | Y | U | U | N |
| Brewer ES et al | 2011 | data ICI | N | Y | na | Y | na | Y | Y | Y | N | N | Y | Y | U | N |
| Kisat M et al | 2011 | data CT, NS | N | Y | Y | na | U | Y | Y | Y | N | N | Y | Y | U | N |
| Abdul Rahman YS et al | 2010 | data CT | N | Y | Y | na | na | Y | Y | Y | N | N | Y | Y | U | N |
| Claudia C et al | 2010 | data ICI | N | Y | na | Y | na | Y | Y | Y | N | N | Y | Y | U | N |
| Fabbri A et al | 2010 | data ICI | N | Y | na | N | na | N | N | Y | N | N | Y | Y | U | N |
| Jacobs B et al | 2010 | data CT, ICI | N | Y | Y | N | na | N | N | Y | N | N | Y | Y | U | N |
| Kotlyar S et al | 2010 | data CT | N | Y | Y | na | na | Y | Y | Y | Y | N | Y | U | U | N |
| Muller B et al | 2010 | data CT | N | Y | U | na | na | Y | Y | Y | N | N | Y | Y | U | N |
| Bouvier D et al | 2009 | data CT, NS | N | Y | Y | na | U | Y | Y | Y | Y | N | Y | U | U | N |
| Morochovic R et al | 2009 | data ICI | N | Y | na | Y | na | Y | Y | Y | Y | N | Y | U | U | N |
| Fabbri A et al | 2008 | data ICI | N | Y | na | Y | na | Y | Y | Y | N | N | Y | Y | U | N |
| Turedi S et al | 2008 | data CT, NS | N | Y | U | na | N | Y | Y | Y | Y | Y | Y | Y | U | N |
| Muller K et al | 2007 | data ICI, NS | N | Y | Y | Y | U | Y | Y | Y | Y | N | Y | U | U | N |
| Saboori M et al | 2007 | data ICI, NS | N | Y | Y | Y | N | Y | Y | Y | Y | N | Y | Y | U | N |
| Smits M et al | 2007 | data ICI | N | Y | na | Y | na | Y | Y | Y | N | N | Y | Y | U | N |
| Bazarian J et al | 2006 | data CT, ICI | N | Y | U | Y | na | Y | Y | Y | Y | N | Y | U | U | N |
| Biberthaler P et al | 2006 | data CT, NS | N | Y | Y | na | U | Y | Y | Y | Y | N | Y | U | U | N |
| Poli-de-Figueiredo LF et al | 2006 | data CT | N | Y | Y | na | na | Y | Y | Y | Y | N | Y | U | U | N |
| Mower WR et al | 2005 | data ICI | N | Y | na | Y | na | Y | Y | Y | Y | N | Y | Y | U | N |
| Munoz-Sanchez MA et al | 2005 | data ICI | N | Y | na | Y | na | Y | Y | Y | N | N | Y | Y | U | N |
| Stiell IG et al | 2005 | data ICI, NS | N | Y | na | N | N | N | U | Y | Y | Y | Y | Y | U | N |
| Abdul Latip LS et al | 2004 | data ICI | N | Y | na | Y | na | Y | Y | Y | N | N | Y | Y | U | N |
| Fabbri A et al | 2004 | data ICI, NS | N | Y | na | N | N | N | N | Y | N | N | Y | Y | U | N |
| Ibanez J et al | 2004 | data ICI | U | N | na | Y | na | Y | Y | Y | Y | N | Y | Y | U | N |
| Nygren de boussard C et al | 2004 | data CT | N | Y | Y | na | na | Y | Y | Y | Y | N | Y | U | U | N |
| Falimirski ME et al | 2003 | data CT | N | Y | U | na | na | Y | Y | Y | N | Y | Y | Y | U | N |
| Mack LR et al | 2003 | data ICI | N | Y | na | Y | na | Y | Y | N | N | N | Y | Y | U | N |
| Biberthaler P et al | 2002 | data CT | N | Y | Y | na | na | Y | Y | Y | Y | N | Y | U | U | N |
| Mussack T et al | 2002 | data CT, ICI, NS | N | Y | Y | Y | N | Y | Y | Y | Y | N | Y | Y | U | N |
| Biberthaler P et al | 2001 | data CT, NS | N | Y | Y | na | U | Y | Y | Y | Y | N | Y | U | U | N |
| Stiell IG et al | 2001 | data CT ICI | N | Y | N | N | na | N | U | Y | Y | Y | Y | Y | U | N |
| Haydel MJ et al | 2000 | data ICI | N | Y | na | Y | na | Y | Y | Y | Y | Y | Y | Y | U | N |
| Ingebrigtsen T et al | 2000 | data CT, ICI, NS | N | Y | Y | Y | N | Y | Y | Y | Y | N | Y | U | U | N |
| Livingston DH et al | 2000 | data ICI | N | Y | na | Y | na | Y | Y | Y | N | N | Y | Y | U | N |
| Vilke GM et al | 2000 | data ICI | N | Y | na | Y | U | Y | Y | Y | N | N | Y | Y | U | N |
| Nagy KK et al | 1999 | data CT | N | Y | U | na | na | Y | Y | Y | N | N | Y | Y | U | N |
| Murshid WR et al | 1998 | data ICI, NS | N | Y | na | N | U | N | N | Y | N | N | Y | Y | U | N |
| Arienta C et al | 1997 | data ICI, NS | U | N | na | N | U | N | N | Y | N | N | Y | Y | U | N |
| Holmes JF et al | 1997 | data ICI, NS | N | Y | na | Y | N | Y | Y | Y | N | Y | Y | Y | U | N |
| Hsiang JNK et al | 1997 | data CT, NS | N | Y | U | na | U | N | N | N | N | N | Y | Y | U | N |
| Miller EC et al | 1997 | data CT | N | Y | Y | na | na | Y | Y | Y | N | Y | Y | Y | U | N |
| Culotta V et al | 1996 | data ICI, NS | N | Y | na | N | U | N | N | Y | N | N | Y | Y | U | N |
| Dunham C et al | 1996 | data ICI | N | Y | na | N | na | N | N | Y | N | N | Y | Y | U | N |
| Gomez PA et al | 1996 | data ICI, NS | N | Y | na | N | N | N | N | Y | N | N | Y | Y | U | N |
| Hung CC et al | 1996 | data ICI | U | N | na | U | na | U | U | N | N | N | Y | Y | U | N |
| Miller EC et al | 1996 | data CT | N | Y | Y | na | na | Y | Y | Y | N | Y | Y | Y | U | N |
| Borczuk P et al | 1995 | data CT | N | Y | U | na | na | Y | Y | Y | N | N | Y | Y | U | N |
| Lee ST et al | 1995 | data ICI | N | Y | na | U | na | U | U | Y | N | N | Y | Y | U | N |
| Moran SG et al | 1994 | data CT | N | Y | N | na | na | N | N | Y | N | N | Y | Y | U | N |
| Murshid WR | 1994 | data ICI; NS | N | Y | na | N | U | N | N | Y | N | N | Y | Y | U | N |
| Duus BR et al | 1993 | data ICI | N | Y | na | N | na | N | N | N | N | N | Y | Y | U | N |
| Jeret JS et al | 1993 | data ICI, NS | N | Y | na | Y | U | Y | Y | Y | N | N | Y | Y | U | N |
| Schynoll W et al | 1993 | data ICI | N | Y | na | Y | na | Y | Y | Y | N | Y | Y | Y | U | N |
| Ando S et al | 1992 | data ICI | N | Y | na | N | na | N | N | Y | N | N | Y | Y | U | N |
| Gutman MB et al | 1992 | data ICI | N | Y | na | Y | na | Y | Y | Y | N | N | Y | Y | U | N |
| Harad FT et al | 1992 | data ICI | N | N | na | Y | na | Y | Y | N | N | N | Y | Y | U | N |
| Mikhail GM et al | 1992 | data ICI, NS | N | Y | na | N | U | N | N | Y | N | N | Y | Y | U | N |
| Nelson JB et al | 1992 | data CT | N | N | U | na | na | Y | Y | N | N | N | Y | Y | U | N |
| Shackford SR et al | 1992 | data ICI, NS | N | Y | na | N | U | N | N | N | Y | N | Y | Y | U | N |
| Stein SC et al | 1992 | data CT,ICI, NS | N | N | Y | Y | U | Y | Y | N | N | N | Y | Y | U | N |
| Livingston DH et al | 1991 | data ICI | N | Y | na | Y | na | Y | Y | Y | N | N | Y | Y | U | N |
| Livingston DH et al | 1991 | data ICI | N | Y | na | N | na | N | N | Y | N | N | Y | Y | U | N |
| Rosenorn J et al | 1991 | data ICI | U | N | na | U | na | U | U | N | N | N | Y | Y | U | N |
| Stein SC et al | 1990 | data CT | N | N | Y | na | na | Y | Y | N | N | N | Y | Y | U | N |
| Teasdale GM et al | 1990 | data NS | N | N | na | na | N | U | U | N | N | N | Y | Y | U | N |
| Feuerman T et al | 1988 | data ICI, NS | U | N | na | N | U | N | N | Y | N | N | Y | Y | U | N |
| Servadei F et al | 1988 | data ICI, NS | N | Y | na | U | N | U | U | Y | N | N | Y | Y | U | N |
| Masters SJ et al | 1987 | data ICI | N | Y | na | U | na | U | U | Y | N | N | Y | Y | U | N |
| Dacey RG et al | 1986 | data CT, ICI, NS | N | Y | N | N | N | N | N | Y | N | N | Y | Y | U | N |
