# Supplementary material for: Scandinavian guidelines for initial management of minimal, mild and moderate head injuries in adults: an evidence and consensus-based update
Source: BMC Med. 2013 Feb 25;11:50. doi: 10.1186/1741-7015-11-50 (PMC3621842; doi:10.1186/1741-7015-11-50)
Supplement: Additional file 4 — Table S4. Evidentiary table of studies with reference to the clinical question: 'Which adult patients with minimal, mild and moderate head injury need in-hospital observation and/or a repeat head CT?'. CT = computed tomography. [file 1741-7015-11-50-S4.DOC]

Additional file 4: Table S4. Evidentiary table of studies with reference to the clinical question: “Which adult patients with minimal, mild and moderate head injury need in-hospital observation and/or a repeat head CT?”. MHI=Minor Head Injury, P=Prospective, R=Retrospective, GCS=Glasgow Coma Scale, HI=Head Injury, CT=Computed Tomography, LOC=Loss of consciousness, PTA=post-traumatic amnesia, GOS=Glasgow Outcome Score, SB=Selection bias, VB=Verification bias.

| **Study** | **Year** | **Design** | **n** | **Age** | **GCS** | **Other inclusion criteria** | **Exclusion criteria** | **Follow-up** | **Evidence level** | **Relevant findings** | **Limitations and comments** |
| --- | --- | --- | --- | --- | --- | --- | --- | --- | --- | --- | --- |
| Tong WS et al | 2012 | R | 498 | All | na | Consecutive HI patients with initial CT within 24 hours | No routine repeat CT ordered | GOS at 6 months post injury | 4 | 139/498 had worsening repeat CT scans. Independant predictors for worse CT scans were factors from the initial CT scan and D-Dimer blood test. Higer age, admission GCS, initial CT, PT, Fibrinogen and D-Dimer were dependant predictors. | SB. No neurosurgery reported. |
| Washington CW et al | 2012 | R | 321 | >17 | 13-15 | Isolated HI with no other injury requiring ICU admission, any ICH on initial CT, initial management non-operative | Patients where iniital management was surgery | GOS | 2 | 19/302 had CT evident injury progression. 4/321 needed neurosurgery, 1 of these had neurological decline. Higher age, anticoagulation and ICH vol>10ml were predictve of CT worsening but only ICH vol was independant. | SB, low risk VB. |
| Menditto VG et al | 2012 | P | 97 | >13 | 14-15 | Any Hi other than superficial face injury, presentation within 48 hours of trauma, warfarin therapy at least 1 week, ISS<15 | Initial CT scan with ICI | Up to 30 days | 2 | 5/87 has intracranial lesions on follow scan, only one of these showed neurological deterioration. 1 of these underwent neurosurgery. 2 addittional patients were readmitted after 2 and 8 days with new CT findings, none of these needed surgery. | Unclear if patient requiring neurosrugery had neurlogical deterioration |
| Connon FF et al | 2011 | P | 591 | >17 | All | Admission for >24 hours following blunt HI, initial CT scan | Patients declared dead within 24 hours, incomplete data or immediate craniotomy/craniectomy | Until discharge | 2 | None of the 156 patients with "routine" repeat CT scans had any change in management. However, 28/149 of CT´s performed for clinical deterioration. 21/156 "routine" repeat CT scans showed radiological deterioration. | SB. Definition of neurosurgical intervention , "change in management" was medical or surgical intervention for ICP treatment. |
| Peck KA et al | 2011 | R | 424 | >14 | na | Blunt HI, preinjury warfarin, clopidogrel, heparin, enoxiparin or didyridamole+asprin | Aspirin alone, warfarin with INR<1.3 | Until discharge | 4 | 4/424 patients had a positive (n=3) or eqvivocal (n=1) repeat CT. All these were minor findings and all patients had no change in neurological examination. | SB, VB. |
| Dalbayrak S et al | 2011 | P | 112 | All | >7 | Hospitalised HI patients with changes between intitial and late CT | GCS<8 | Medical records | 4 | 103/112 had worsening CT findings and neurological status deteriorated in only 30% of these. 46/112 needed neurosurgery and neurological status was stable in 50% of these. | SB |
| Schaller et al | 2010 | R | 110 | All | 13-15 | HI with localised epidural, subdura and subarachnoidal heamatomas <5mm in diameter | Multiple bleeds, coagulopathy/anticoagulantia, anti-platelet medication, intoxicaiton, multiple injuries, no home observer and patients who lived >1 hour from the site | Medical records | 3 | All patients maintained/improved in GCS and clinical status over 24 hours. No need for repeat CT in any patients. | SB |
| Alahmadi H et al | 2010 | R | 98 | 17-86 | All | HI with initial CT showing contusion, initial management conservative and at least 1 repeat CT scan | Craniotomi after initial scan, patients who did npt recive repeat CT or neurosurgery and patietns discharged directly. | Until discharge | 4 | 44/98 has rediographic progression and 19/98 has neurosurgery. Referring to initial GCS scores, 11% of GCS 14-15, 32% of GCS 9-13 and 58% of GCS 3-8 needed delayed surgery. | SB. Only contusions included. |
| Bee TK et al | 2009 | R | 207 | All | 14-15 | LOC and/or retrograde amnesia, intracererbak injury on initial CT | Skull fractures, facial fractures needing urgernt repair, direct neurosurgery, other injuries requiring ICU minitoring | No | 2 | 58/207 showed worsening on repeat CT. 18/207 needed neurosurgical intervention, 5 of these had no neurological decline (all subdrual haematomas). | SB. Unclear indication for neurosurgery in asymptomatic patients. |
| Kaen A et al | 2009 | P | 137 | >16 | 14-15 | HI and treatement with heparin or warfarin | No | Until discharge | 4 | 2/137 patients has positive repeat CT scans and none had neurological deterioration or neurosurgery | Neurological deterioration defined as change in initial GCS with or without other symtoms |
| Tauber et al | 2009 | P | 100 | >64 | 15 | Regular los-dose aspirin therapy, initial negative CT, no hypertenisve irregularities | Anticoagulants, moderate-severe HI. Patients with pathology on initial CT | Until discharge | 4 | 4/100 has positive repeat CT scans, all without neurological deterioration. 1 patient died (age 84) after neurological deterioration and 1 patient need neurosurgery but first after neurological deterioration. | SB. Unclear if neurological deterioration could have been used as test for repeat CT. |
| Turedi S et al | 2008 | P | 240 | All | 13-15 | Blunt HI, LOC < 15 min or post-traumatic amnesia <1 hr | No | No | 2 | Repeat CT scans in 120 patients with high risk criteria (GCS 14-15 and LOC, amnesia, vomiting, suspected skull fracture, multiple trauma, severe/increasing headache, aymmetric pupils, focal neurology, post-traumatic seizures or anticoagulant/coagulopathy) showed abnormalities in 3 and none of these needed neurosurgery. |  |
| Brown CV et al | 2007 | P | 274 | All | All | Blunt HI and ICH on initial CT | Immediate neurosurgery and death within 24 hours | Until discharge | 2 | 163/274 underwent repeat CT scans. 17/45 of repeat CT scans for neurological change led to a medical or surgical intervention vs 2/196 routine scans led to an intervention, The 2 cases of intervention after routine scans were in patients with severe head injury (GCS <9). | VB |
| Sifri ZC et al | 2006 | P | 130 | >17 | 13-15 | HI and intracranial bleed or contusion on initial CT | Prior brain surgery or cerebral pathology, chronic neurological condition, spinal cord injury, coagulopathy, anticoagulation, immediate or planned neurosurgery after the initial CT and patients who never had a follow-up CT | GOS in discharge | 4 | 99/130 patients had normal neurological findings at repeat CT and none had neurosurgery or deterioration. 31/130 had abnomral neurological findings and 2 needed immediate neurosurgery. In patients with normal neurological exam, no change or improvement in 87% of repeat CT scans but no change in management. Fior the 12 CT´s that were worse, these patients all had favourable outcome. | SB. CT change classified as improved, worse or unchanged by neurosurgical team. Neurosurgical intervention defined as craniotomy or ICP monitor. |
| Itshayek E et al | 2006 | R | 4 | 65-86 | 15 | HI patients with anticoagulation with normal initial CT and delayed acute subdural haematoma | No | GOS up to 26 months | 4 | 4 patients with minimal HI (GCS 15, no LOC/amnesia) and normal initial CT all showed delayed subdrual haematoma after 9 hours to 3 days post-trauma. | SB. Case series. |
| Velmahos GC et al | 2006 | R | 179 | All | 13-15 | LOC, short-term amnesia, headache, emesis or dizziness | No routine repeat CT ordered | Medical records | 4 | 37/179 patients had progress of CT injury and 7 of these needed medical or neurosurgical intervention. All of these 7 patients had clinical deterioration before repeat CT. Lower GCS and higher age were predictors of worse repeat CT. | SB. NS = medical or neurosurgical intervention |
| Sifri ZC et al | 2004 | R | 202 | >15 | 14-15 | HI with LOC/amnesia and positive initial CT scan | History of brain injury or coagulopathy. Patients who required immediate neurosurgery after initial CT | No | 4 | 22/151 patients with normal/improved neurological examination at 24 hours had worse CT scans, none (of the 151) needed surgery. 18/51 patients with abnormal/worsening neurological examination at 24 hours has worse CT scans, 5 needed surgery. | SB |
| Fainardi E et al | 2004 | R | 141 | All | All | Hi with traumatic subarchnoid haemorrahge on initial CT | Brain death on admission, hypotension due to extracranial injuries and penetrating injuries not due to traffic accidents | GOS at 6 months post injury | 2 | 83 patients had worse repeat CT. 30 of these patients had GCS 14-15 , 32 had GCS 9-13 and 21 had GCS 3-8. 38 patients had significant Ct worsening (worse CT and change in Marshall category). Of these, 7 were GCS 14-15, 18 were GCS 9-13 and 13 were GCS 3-8. | SB. Only patients with evidence of traumatic subarachnoid blood on initial CT. |
| Brown CV et al | 2004 | P | 100 | >17 | All | Consecutive blunt HI patients with abnormal initial CT | Isolated skull fracture/pneumocephalus. Patients who underwent immediate craniotomy and patients declared brain dead or died. | Until discharge | 2 | 68 patients underwent 90 repeat CT scans. 81/90 CT scans were routine, none of these led to any intervention. 9/81 ST scans were due to neurological deterioration and 3 of these needed intervention. | Intervention defined as medical or surgical |
| Livingston DH et al | 2000 | P | 2152 | >15 | 14-15 | LOC or posttraumatic amnesia | GCS<14, focal neurological deficit, open skull fracture, clinical basilar skull fracture, anticoagulantia, cirrhosis, emergency operation before CT, severe heart disease, bleeding disorder, low platelet count, renal dialysis | 4-8 hours, 20 hours and at discharge | 3 | Patients with GCS 14-15 and LOC/posttraumatic amnesia with an initial normal CT scan kan be safely discharged in absence of persistent neurological findings and other body system injuries | SB. Definition of neurosurgical intervention includes intubation, anticonvulsives and anti-eodema treatment |
| Nagy KK et al | 1999 | P | 1170 | All | 15 | Blunt HI with LOC/amnesia | No | Short-term hospital follow-up | 2 | Admission of patients with GCS 15 and LOC/amnesia and with normal initial CT results is unneccessary | SB |
