# Supplementary material for: Scandinavian guidelines for initial management of minimal, mild and moderate head injuries in adults: an evidence and consensus-based update
Source: BMC Med. 2013 Feb 25;11:50. doi: 10.1186/1741-7015-11-50 (PMC3621842; doi:10.1186/1741-7015-11-50)
Supplement: Additional file 5 — Table S5. Modified Quality Assessment of Diagnostic Accuracy Studies (QUADAS) grading of studies referring to the clinical question: 'Which adult patients with minimal, mild and moderate head injury need in-hospital observation and/or a repeat head CT?'. CT = computed tomography. [file 1741-7015-11-50-S5.DOC]

Additional file 5: Table S5. Modified QUADAS grading of studies referring to the clinical question: “Which adult patients with minimal, mild and moderate head injury need in-hospital observation and/or a repeat head CT?”. CT=Computed Tomography, ICI=Intracranial injury, NS=Neurosurgery, Y=Yes, N=no, U=Unknown, na=not applicable.

| **Study** | **Year** | **Data** | **Low risk of selection bias/spectrum of patients representative** | **Selection criteria described well** | **Acceptable reference test, CT** | **Acceptable reference test, ICI** | **Acceptable reference test, NS** | **Did all patients recieve reference test** | **Did all patients recieve same reference test** | **Index test described well** | **Reference test descibed well** | **Index test blinded to reference test** | **Reference test blinded to index test** | **Same clinical data as in practice** | **Uninterpretable data reported** | **Withdrawals explained** |
| --- | --- | --- | --- | --- | --- | --- | --- | --- | --- | --- | --- | --- | --- | --- | --- | --- |
| Tong WS et al | 2012 | ICI | U | U | na | Y | na | Y | Y | Y | N | Y | N | U | N | N |
| Washington CW et al | 2012 | ICI, NS | N | Y | na | Y | Y | N | Y | Y | N | Y | Y | U | N | N |
| Menditto VG et al | 2012 | ICI, NS | Y | Y | na | Y | Y | Y | Y | Y | N | Y | N | U | N | N |
| Connon FF et al | 2011 | CT, NS | N | Y | Y | na | Y | N | Y | U | N | Y | N | U | N | N |
| Peck KA et al | 2011 | ICI, NS | N | Y | na | Y | U | N | N | N | N | Y | Y | U | N | Y |
| Dalbayrak S et al | 2011 | CT, NS | N | U | Y | na | Y | Y | Y | Y | N | Y | N | U | N | N |
| Schaller et al | 2010 | CT | N | Y | U | na | na | N | Y | N | N | Y | N | U | N | N |
| Alahmadi H et al | 2010 | ICI, NS | N | Y | na | Y | Y | Y | Y | N | N | Y | N | Y | N | N |
| Bee TK et al | 2009 | ICI, NS | N | Y | na | Y | Y | Y | Y | Y | N | Y | N | Y | N | N |
| Kaen A et al | 2009 | ICI, NS | Y | Y | na | Y | U | Y | Y | Y | N | Y | N | U | N | N |
| Tauber et al | 2009 | ICI, NS | N | Y | na | Y | Y | Y | Y | Y | Y | Y | Y | Y | N | N |
| Turedi S et al | 2008 | CT, NS | N | Y | U | na | N | Y | Y | Y | Y | Y | Y | Y | U | N |
| Brown CV et al | 2007 | ICI, NS | Y | Y | na | Y | Y | N | Y | Y | N | Y | N | Y | N | N |
| Sifri ZC et al | 2006 | ICI, NS | N | Y | na | Y | Y | Y | Y | Y | N | Y | N | Y | N | Y |
| Itshayek E et al | 2006 | ICI, NS | N | N | na | Y | Y | Y | Y | Y | Y | Y | N | Y | N | N |
| Velmahos GC et al | 2006 | ICI, NS | N | Y | na | Y | Y | Y | Y | Y | Y | Y | N | Y | N | U |
| Sifri ZC et al | 2004 | ICI, NS | N | Y | na | Y | Y | Y | Y | Y | N | Y | N | Y | N | Y |
| Fainardi E et al | 2004 | ICI | N | Y | na | Y | na | Y | Y | Y | N | Y | N | Y | N | Y |
| Brown CV et al | 2004 | ICI, NS | Y | Y | na | Y | Y | N | Y | Y | N | Y | N | Y | N | Y |
| Livingston DH et al | 2000 | NS | N | Y | na | na | Y | Y | Y | N | Y | Y | N | Y | Y | Y |
| Nagy KK et al | 1999 | NS | N | N | na | na | U | U | Y | N | N | Y | N | U | N | N |
