# Supplementary material for: Scandinavian guidelines for initial management of minimal, mild and moderate head injuries in adults: an evidence and consensus-based update
Source: BMC Med. 2013 Feb 25;11:50. doi: 10.1186/1741-7015-11-50 (PMC3621842; doi:10.1186/1741-7015-11-50)
Supplement: Additional file 6 — Figure S1. Discharge advice for adults following minimal, mild and moderate head injury. [file 1741-7015-11-50-S6.DOC]

Discharge advice for a person who has had a head injury

A doctor has carefully checked you after your head injury. It is now safe for you to leave the hospital but you should not be alone for the next 24 hours. Someone living with you should check on you one time during the night.

**Things you shouldn’t worry about**

You may feel some symptoms over the next few days that should go away in the next 2 weeks. These include a mild headache, problems concentrating or with your memory (forgetfulness), feeling sick (without vomiting), dizziness, feeling irritated (having a bad temper), abnormal tiredness or problems sleeping. If you feel very concerned about any of these symptoms, or if they do not go away after 2 weeks, you should see your family doctor to talk about them. We also recommend that you see your family-doctor about your ability to drive a motor vehicle if you experience any of these problems.

**Things that you should NOT do**

- **DO NOT** take any alcohol or drugs.
- **DO NOT** take sleeping pills, sedatives or tranquilisers unless they are prescribed by a doctor.
- **DO NOT** do any contact sport (for example, martial arts, rugby, ice hockey or football) or horseback riding for at least 1-2 weeks without talking to your doctor first.

**Long-term problems**

Most people get better quickly and do not have long-term problems. However, some people have problems later on, after weeks or even months. If you start to feel that things are not quite right (not feeling yourself), then please contact your family doctor so that he or she can check to make sure you are recovering properly.

**Contact emergency medical services if other alarming symptoms occur (such as vomiting, severe headache, confusion and/or problems keeping awake).**

***Contact information:***
