# Supplementary material for: Scandinavian guidelines for initial management of minimal, mild and moderate head injuries in adults: an evidence and consensus-based update
Source: BMC Med. 2013 Feb 25;11:50. doi: 10.1186/1741-7015-11-50 (PMC3621842; doi:10.1186/1741-7015-11-50)
Supplement: Additional file 7 — Figure S2. Final management guidelines for adults following minimal, mild and moderate head injury (including help sheet). [file 1741-7015-11-50-S7.DOC]

Discharge with oral and written instructions

Some patients may need admission for reasons other than their head injury

**Yes**

All adult patients with minimal, mild and moderate head injury (GCS 9-15) within 24 hours of injury

**Minimal**

GCS 15

Admission for observation ≥24 hours

Consider consultation with neurosurgeon

Repeat CT if neurological and/or GCS (≥2 points) deterioration

**No**

CT

Consider admission for observation ≥12 hours after injury as an alternative option

CT

Consider admission for observation ≥12 hours after injury as an alternative option

**Yes**

**Mild**

medium-risk

GCS 14-15 *and*

BOTH age ≥65 years *AND* anti-platelet medication

**No**

CT

**Yes**

**Yes**

S100B

If <6 hrs after injury, sample serum for S100B analysis

If ≥6 hrs, extracranial injury or if S100B is unavailable, do CT

**Yes**

**Mild**

high-risk

GCS 14-15 *and*

Posttraumatic seizures

Focal neurological deficits

Clinical signs of depressed or basal skull fracture

Shunt-treated hydrocephalus

Therapeutic anticoagulation or coagulation disorders

**Mild**

low-risk

GCS 14

*or*

GCS 15 *and*

Suspected/confirmed loss of consciousness

Repeated vomiting (≥2 episodes)

**Moderate**

GCS 9-13

**No**

**No**

*See help sheet for explanations and more details*

**< 0.10**

**µg/L**

**CT abnormal**

**CT**

**normal**

**CT**

**abnormal**

**CT**

**normal**

**or**

**abnormal**

**CT**

**normal**

**≥ 0.10**

**µg/L**

***help sheet***

**Scandinavian guidelines for initial management of adult patients with minimal, mild and moderate head injury**

**General points**

This guideline is a based upon evidence-derived recommendations and consensus aspects from the Scandinavian Neurotrauma Committee (SNC) working group. Being a guideline, it should not override clinical judgement and may be super-seeded if necessary.

GCS scores should be noted as the score after resuscitation. A thorough clinical exam should include aspects so that the various risk factors may be adequately assessed.

**Minimal**

These patients are GCS 15 and have none of the risk factors mentioned in the other boxes. These patients have a very low risk of serious intracranial complication and can be discharged from the hospital without a computed tomography (CT) scan.

**Mild. low-risk**

These patients are GCS 14 without risk factors or, alternatively, are GCS 15 and have suspected or confirmed loss of consciousness (i.e. the patient cannot clearly deny loss of consciousness) or repeated vomiting (at least two episodes). If less than 6 hours have passed since the trauma, and S100B analysis is readily available, sample serum. If S100B is <0.10 µg/L, the patient has a very low risk of serious intracranial complication and can be discharged. If S100B is ≥0.10 µg/L, a CT scan should be done. Similarly, if more than 6 hours have passed, the patient has significant extracranial injuries (for instance large bone fractures may result in elevated S100B levels) or S100B analysis is unavailable, a CT scan is instead indicated. If the CT is normal, the patient can be discharged.

**Mild, medium-risk**

These patients are GCS 14-15 and are 65 years or older whilst also taking anti-platelet medication (such as acetyl acid derivatives (aspirin), clopidogrel, ticlopidine and dipyridamole). These patients have a moderate risk of intracranial complication and should have a CT scan and, if normal, can be discharged.

**Mild, high risk**

These patients are GCS 14-15 and have a intraventricular shunt, have had post-traumatic seizures, have clinical signs of depressed skull fracture (palpable depression or abnormal discontinuity of skull) or basal skull fracture (raccoon eyes, Battle’s sign, hemotympanum , cerebrospinal fluid rhinorrhea), have focal neurological deficits (function loss (for instance motor skills) from a specific part of the body (for instance face, arm or leg) suggestive of cerebral dysfunction), are taking anticoagulation medication (coumadin/warfarin, low molecular-weight heparins, dabigatran or other pharmacologic anticoagulation) or have known coagulation disorders (such as haemophilia, thrombocytopenia or lever cirrhosis with pathological INR (>1.5)). These patients are relatively uncommon but have a relatively high risk of intracranial complication. They should have a CT and also be admitted for close observation for at least 24 hours, even if the CT is normal.

**Moderate**

These patients are GCS 9-13. They should have a CT and also be admitted for close observation for at least 24 hours, even if the CT is normal.

---------------------------------------------------------------------------------------------------------------------------------------

**Admission**

CT is strongly recommended as the primary management routine. If CT is unavailable or logistically difficult, some patients may be admitted for close neurological observation for at least 12 hours after injury.

Patients with high-risk mild and moderate head injury should be admitted, irrespective of CT findings, for at least 24 hours. Observation including GCS, pupil size/reactivity, a simplified neurological exam, blood pressure, pulse rate, oxygen saturation and respiration rate should be performed every 15 minutes for the first 4 hours after injury, every 30 minutes for the following 4 hours and at least every hour hereafter.

Some patients with minimal head injury or normal CT/serum S100B following mild head injury, where discharge is recommended, may need admission for other reasons than head injury (such as elderly patients without sufficient help at home, patients with other injuries or patients with heavy intoxication). Since these patients have a very low risk of intracranial injury, they do not need the extensive observation routine mentioned above.

**CT**

A non-contrast CT, according to local radiology routines, should be done as quickly as possible, with a greater urgency for more severe head injuries according to the above. The patient should be adequately monitored when waiting for the CT scan. When CT is abnormal, consider contacting a neurosurgeon or neurotrauma centre for advice concerning further management.

**Repeat CT**

Routine repeat CT is not recommended. However, a repeat CT should be done immediately in patients with deterioration in GCS (≥2 points) or new/progressive neurological deficits.

**Discharge**

ALL patients with head injury should receive oral and written instructions at discharge (see separate form). These give general advice concerning their head injury.
